# A systems-biological study on the identification of safe and effective molecular targets for the reduction of ultraviolet B-induced skin pigmentation

**DOI:** 10.1038/srep10305

**Published:** 2015-05-18

**Authors:** Ho-Sung Lee, Myeong-Jin Goh, Junil Kim, Tae-Jun Choi, Hae Kwang Lee, Yong Joo Na, Kwang-Hyun Cho

**Affiliations:** 1 Laboratory for Systems Biology and Bio-Inspired Engineering, Department of Bio and Brain Engineering, Korea Advanced Institute of Science and Technology, Daejeon, 305-701, Republic of Korea; 2 Graduate School of Medical Science and Engineering, Korea Advanced Institute of Science and Technology, Daejeon, 305-701, Republic of Korea; 3 Skin Research Institute, Amorepacific R&D center, Gyeonggi-do, 446-729, Republic of Korea

## Abstract

Melanogenesis is the process of melanin synthesis through keratinocytes-melanocytes interaction, which is triggered by the damaging effect of ultraviolet-B (UVB) rays. It is known that melanogenesis influences diverse cellular responses, including cell survival and apoptosis, via complex mechanisms of feedback and crosstalk. Therefore, an attempt to suppress melanin production by modulating the melanogenesis pathway may induce perturbations in the apoptotic balance of the cells in response to UVB irradiation, which results in various skin diseases such as melasma, vitiligo, and skin cancer. To identify such appropriate target strategies for the reduction of UVB-induced melanin synthesis, we reconstructed the melanogenesis signaling network and developed a Boolean network model. Mathematical simulations of the melanogenesis network model revealed that the inhibition of beta-catenin in the melanocytes effectively reduce melanin production while having minimal influence on the apoptotic balance of the cells. Exposing cells to a beta-catenin inhibitor decreased pigmentation but did not significantly change the B-cell Chronic lymphocytic leukemia/lymphoma 2 expression, a potent regulator of apoptotic balance. Thus, our systems analysis suggests that the inhibition of beta-catenin may be the most appropriate target strategy for the reduction of UVB-induced skin pigmentation.

Melanogenesis, the process of skin color formation, is controlled by a complex molecular regulatory network embedded in the keratinocytes and melanocytes, which results in the skin and hair pigmentation^1^,^2^,^3^. When skin is exposed to ultraviolet-B (UVB) irradiation, keratinocytes excessively synthesize various biochemical factors such as alpha-Melanocyte-stimulating hormone (a-MSH), endothelin-1 (ET-1), stem cell factor (SCF), and prostaglandin E2 (PGE2)^4^. These keratinocytes-derived factors are transported to melanocytes in a paracrine manner and induce Microphthalmia-associated transcription factor (MITF) activation through a series of signaling events, which results in melanin production via activation of tyrosinase-related proteins^1^,^2^,^3^. Synthesized melanin is then transferred to the neighboring epidermal keratinocytes to protect cells from UVB-induced DNA damage response^1^,^2^,^3^.

The melanogenesis and the apoptotic pathways share some of their downstream signaling molecules, which are interlinked with each other through complex mechanisms of feedback and crosstalk^1^,^2^,^3^. For this reason, the paracrine factors produced in response to UVB irradiation not only regulate melanogenesis, but can also affect pathways that are pivotal for the coordination of cell survival and apoptosis. Therefore, an attempt of skin color control by modulating the melanogenesis pathway may induce perturbations in the apoptotic balance of the cells, which can cause various skin diseases such as melasma, vitiligo, and skin cancer^3^,^5^,^6^,^7^. To overcome such biological complexity and investigate appropriate target strategy for the reduction of UVB-induced skin pigmentation, we have developed a novel and comprehensive mathematical model of the melanogenesis network by integrating all available experimental results that are closely associated with the melanin synthesis under UVB irradiation. Mathematical simulations and biochemical experiments revealed that the inhibition of beta-catenin, a transcriptional activator of MITF^8^, can effectively reduce the melanin production while minimally affecting the B-cell Chronic lymphocytic leukemia/lymphoma 2 (Bcl-2) expression, an important regulator of the apoptotic balance of the cells^9^,^10^,^11^. Our findings highlight the significance of network-level analyses for understanding bio-molecular regulatory mechanisms, and finding an optimal target and its intervention strategy to reduce UVB-induced melanin synthesis.

## Results

### A mathematical model of the melanogenesis network

**Figure 1:**
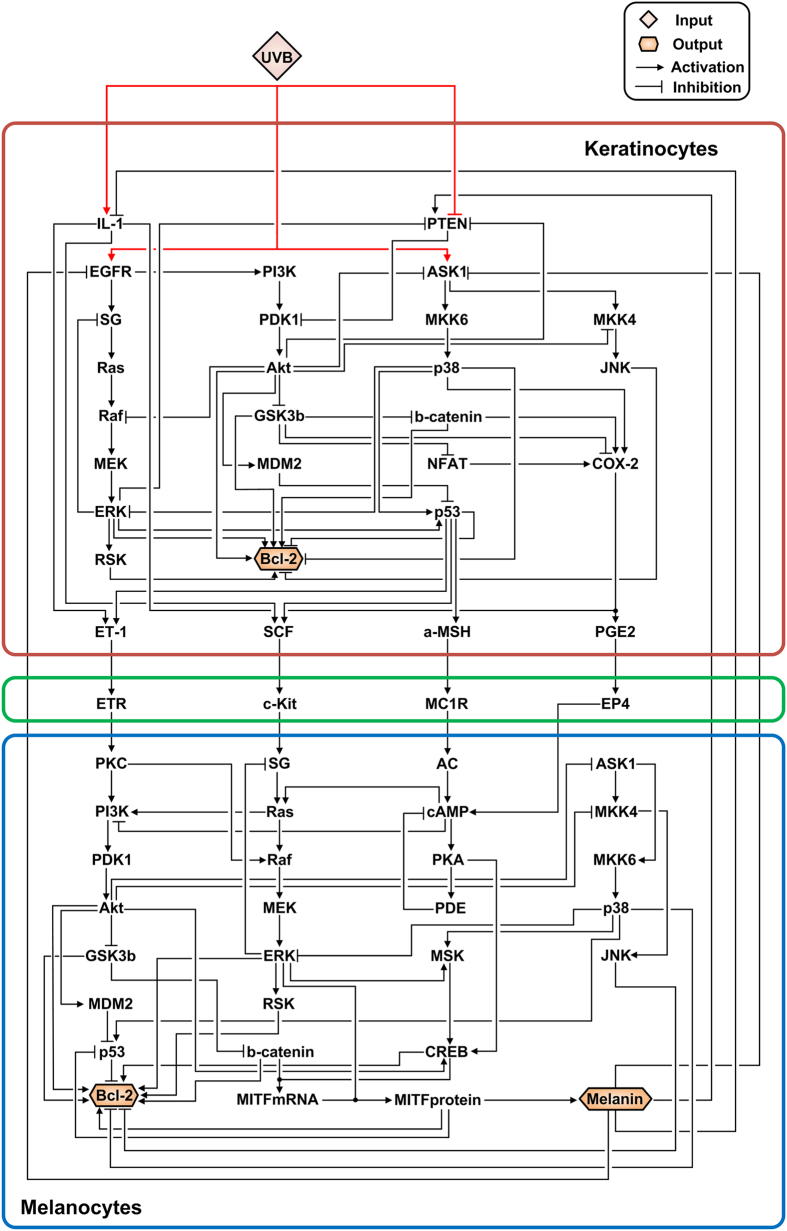
A schematic diagram of the melanogenesis network. The melanogenesis network comprises two major signaling modules: the keratinocytes (red square) and the melanocytes (blue square) modules. The network consists of 62 nodes and 113 links; 80 links are activating (pointed arrows) and 33 are inhibiting (blunted arrows). Among the 62 nodes, there are one external-input node (UVB) and three output nodes (melanin, Bcl-2 in the keratinocytes, and Bcl-2 in the melanocytes) (see Supplementary Materials). IL-1, interleukin 1; PTEN, phosphatase and tensin homolog; EGFR, epidermal growth factor receptor; PI3K, phosphatidylinositol 3-kinase; ASK1, apoptosis signal-regulating kinase 1; SG, the growth factor receptor**–**bound protein 2 (Grb2) and Son of Sevenless (SOS) complex; PDK1, phosphoinositide-dependent kinase 1; MKK6, mitogen-activated protein kinase (MAPK) 6; MKK4, mitogen-activated protein kinase (MAPK) 4; JNK, c-Jun N-terminal kinase; GSK3b, Glycogen synthase kinase-3 beta; b-catenin, beta-catenin; ERK, extracellular signal-regulated kinase; MEK, MAPK/ERK kinase; MDM2, mouse double minute 2 homolog; NFAT, nuclear factor of activated T-cells; COX-2, cyclooxygenase-2; RSK, ribosomal s6 kinase; ETR, endothelin receptor; MC1R, melanocortin 1 receptor; EP4, prostaglandin E receptor 4; PKC, protein kinase C; AC, adenylyl cyclase; cAMP, cyclic adenosine monophosphate; PKA, protein kinase A; PDE, phosphodiesterase; MSK, mitogen- and stress-activated kinase and CREB, cAMP response element-binding protein.

The aim of this study was to investigate the appropriate target strategies for the reduction of UVB-induced skin pigmentation. For this purpose, we first reconstructed the melanogenesis network with the focus on the paracrine factor-mediated signaling pathway and the MITF-related pathway since these two pathways are known to be most crucial in skin pigmentation^2^. We integrated all relevant information on individual key proteins and their interactions through an extensive survey of literatures (see Supplementary Materials). The reconstructed melanogenesis network comprises two major modules, keratinocytes and melanocytes, which are the most important cell types in UVB-induced melanin synthesis^4^. The network consists of 62 nodes and 113 directed links, and contains one external-input node, UVB, which provides a potent extracellular cue for melanogenesis (Fig. S1), and three output nodes (Bcl-2 of keratinocytes, Bcl-2 of melanocytes, and melanin) (Fig. 1 and Table S1). Bcl-2 is a key member of the anti-apoptotic Bcl-2 family and is a potent inhibitor of apoptotic cell death^12^. It inhibits the mitochondrial death signaling and blocks the redistribution of cytochrome c which is released in early stages of apoptosis^13^. Previous studies have suggested that Bcl-2 is a potent regulator of cell fate determination in keratinocytes and melanocytes and that its increased or decreased expression level regulates the apoptotic response upon UVB irradiation^9^,^14^,^15^,^16^,^17^.

Due to the limited information on mechanistic details in the reaction kinetics of each biochemical interaction, continuous, detailed differential equation-based models face difficulties in obtaining biologically reliable parameter estimates^18^. To avoid the problem of parameterization on which detailed models are dependent, parameter-free discrete logic-based models have been applied to explore generic properties of complex biomolecular regulatory networks^18^,^19^,^20^,^21^. Therefore, we established a large-scale discrete Boolean network model of the melanogenesis network based on the mechanistic information about the activation and/or inhibition of each signaling protein (see Supplementary Materials and Table S2). In the Boolean network model, the state value of each node represents its activity, and is discretely represented as either ‘0’ for an inactive or ‘1’ for an active state. Logic tables (representing an instruction set) were created for each node based on experimental evidence obtained from literatures (see Supplementary Materials and Table S2).

**Figure 2:**
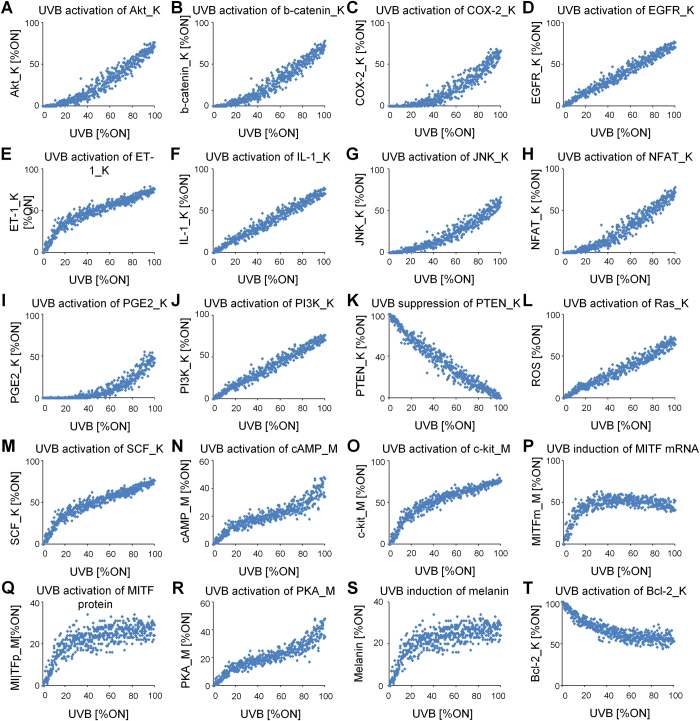
Qualitative, individual input–output relationships in the Boolean model of melanogenesis network. ‘K’ denotes the nodes of keratinocytes and ‘M’ denotes the nodes of melanocytes. (**A**) Positive relationship between UVB and Akt_K activation^22^. Positive relationship between UVB and beta-catenin_K expression^23^. (**C**) Positive relationship between UVB and COX-2_K expression^24^. (**D**) Positive relationship between UVB and EGFR activation_K^25^. (**E**) Positive relationship between UVB and ET-1_K expression^26^. (**F**) Positive relationship between UVB and IL-1_K expression^27^. (**G**) Positive relationship between UVB and JNK_K activation^28^. (**H**) Positive relationship between UVB and NFAT_K activation^29^. (**I**) Positive relationship between UVB and PGE2_K activation^30^. (**J**) Positive relationship between UVB and PI3K_K activation^31^. (**K**) Negative relationship between UVB and PTEN_K activation^22^. (**L**) Positive relationship between UVB and Ras_K activation^32^. (**M**) Positive relationship between UVB and SCF_K activation^33^. (**N**) Positive relationship between UVB and cAMP_M activation^34^. (**O**) Positive relationship between UVB and c-kit_M activation^35^. (**P**) Positive relationship between UVB and induction of MITFmRNA_M^2^. (**P**) Positive relationship between UVB and induction of MITFprotein_M^2^ (**R**) Positive relationship between UVB and PKA_M activation^36^. (**S**) Positive relationship between UVB and melanin synthesis^37^,^38^. (**T**) Negative relationship between UVB and Bcl-2_K expression^39^,^40^. Note that the dose–response curves presented here are intended to demonstrate how the melanogenesis network model qualitatively reproduces the referenced input–output relationships over a wide range of input signal. The simulations were performed repetitively (n = 10) at 2% noise to the input (see Materials and Methods).

To examine the model’s ability to reproduce biological properties of the actual melanogenesis signaling network, qualitative input–output *in silico* simulations were performed with the intensity of the input of interest (UVB) varying from 0% to 100% (see Materials and Methods). The results show that the melanogenesis network model captures qualitative features of the known biological activities of the species in the system^2^,^22^,^23^,^24^,^25^,^26^,^27^,^28^,^29^,^30^,^31^,^32^,^33^,^34^,^35^,^36^,^37^,^38^,^39^,^40^ (Fig.2).

### Identification of safe and effective target strategies for the reduction of UVB-induced skin pigmentation

To identify such safe and effective strategies for the reduction of UVB-induced skin pigmentation, we performed *in silico* node control analysis under UVB irradiation by pinning the state value of each internal regulatory node to either ‘0’ for inhibition or ‘1’ for constitutive activation. The perturbation effect of the node control of each internal regulatory node was measured by the amount of changes in the activities of three output nodes, melanin, Bcl-2_K (‘K’ denotes the nodes of keratinocytes), and Bcl-2_M (‘M’ denotes the nodes of melanocytes) (Table S3) (see Materials and Methods). As a result, node controls shown in Table 1 were found to be potentially effective target strategies for reducing the UVB-induced skin pigmentation since these strategies substantially reduced (<−90%) the activity of node ‘melanin’ in response to UVB irradiation. Most prominent among these potentially effective target strategies was the inhibition of b-catenin_M which effectively reduced the activity of melanin while having minimal influence on the activities of both ‘Bcl-2_K’ and ‘Bcl-2_M’ in the model simulation.

Previous studies have shown that complex cellular signaling networks can exhibit ordered (stable) dynamics, raising the possibility that cellular behaviors or phenotypes can be represented as high-dimensional attractor states^41^,^42^,^43^. An attractor is a mathematical concept that refers to a stable steady-state to which a dynamical system, in this case a cellular signaling network, tends to approach over time^43^,^44^. Based on the concept of an attractor, the dynamic behavior of a cellular signaling network can be mapped into an attractor landscape. Each point in the landscape corresponds to a particular network state, defined by the profile of the activation state of all the nodes in the network^45^. The state space of a Boolean network with *N* nodes comprises 2^*N*^ different states and the state transition dynamics are characterized by sequential state flows, eventually converging towards attractors^46^. Such attractors are particular subsets of states, either a fixed point (a point attractor) representing a single network state, or a subset of network states constituting a limit cycle of period *p* (a cyclic attractor), comprised by *p* states sequentially visited by the network dynamics^47^. The set of all initial states that converge towards a given attractor is called the basin of attraction^48^.

To further assess the appropriateness of the potentially effective target strategies for the reduction of UVB-induced skin pigmentation, we applied the state space approach to our melanogenesis network model, which is a useful representation of dynamic behavior of cellular systems^41^,^42^,^43^. The attractors of melanogenesis network were identified from Boolean simulations using a number of randomly sampled initial conditions, or network states. Note that the distributions of estimated (relative) basin sizes of the attractors are very similar regardless of the sampling number (Fig. S2). Distinct attractors of the melanogenesis network can be classified into groups characterized by specific cellular behaviors or phenotypes. The phenotype pigmentation, keratinocytes survival, or melanocytes survival was defined as the corresponding attractor of which ‘melanin’, ‘Bcl-2_K’, or ‘Bcl2-M’, respectively, is ‘ON’ at least once in its cyclic state transitions.

**Figure 3:**
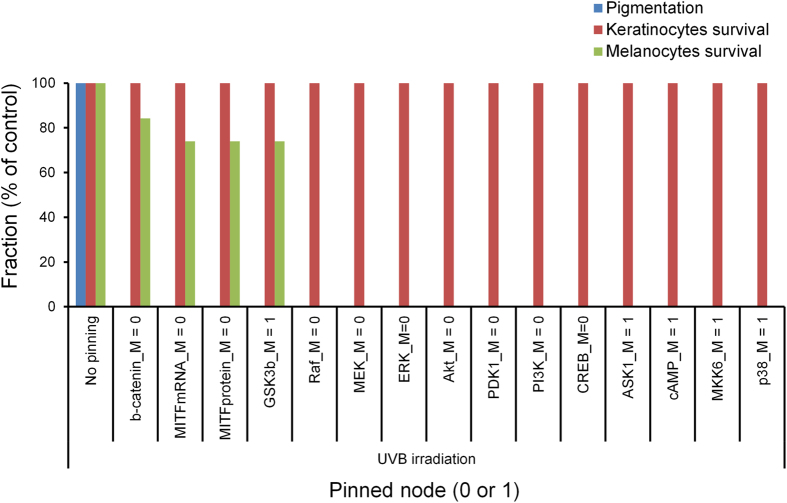
Network behavior in response to potentially effective target strategies under UVB irradiation. Fraction of initial states evolving into pigmentation, keratinocytes survival, or melanocytes survival attractors (phenotypes) in response to potentially effective target strategies under UVB irradiation.

To investigate the influence of the potentially effective target strategies on the cellular phenotype, we performed Boolean simulations under persistent UVB stimulation while pinning the state of each potentially effective target to either ‘0’ for inhibition or ‘1’ for constitutive activation and observed the network behavior. Interestingly, the results of state-space analysis also highlighted the appropriateness of the inhibition of b-catenin_M for the reduction of UVB-induced skin pigmentation. All the potentially effective target strategies significantly reduced the fraction of initial states evolving into the pigmentation attractor (phenotype). Among those strategies, the inhibition of b-catenin_M had minimal influence on the fraction of initial states evolving into the attractors for the keratinocytes survival or the melanocytes survival (Fig. 3), which suggests its safety for use. Taken together, the results indicate that the inhibition of b-catenin_M may be the most appropriate target strategy for the reduction of UVB-induced melanin synthesis.

### Biochemical validation of predictions from the melanogenesis network model

**Figure 4:**
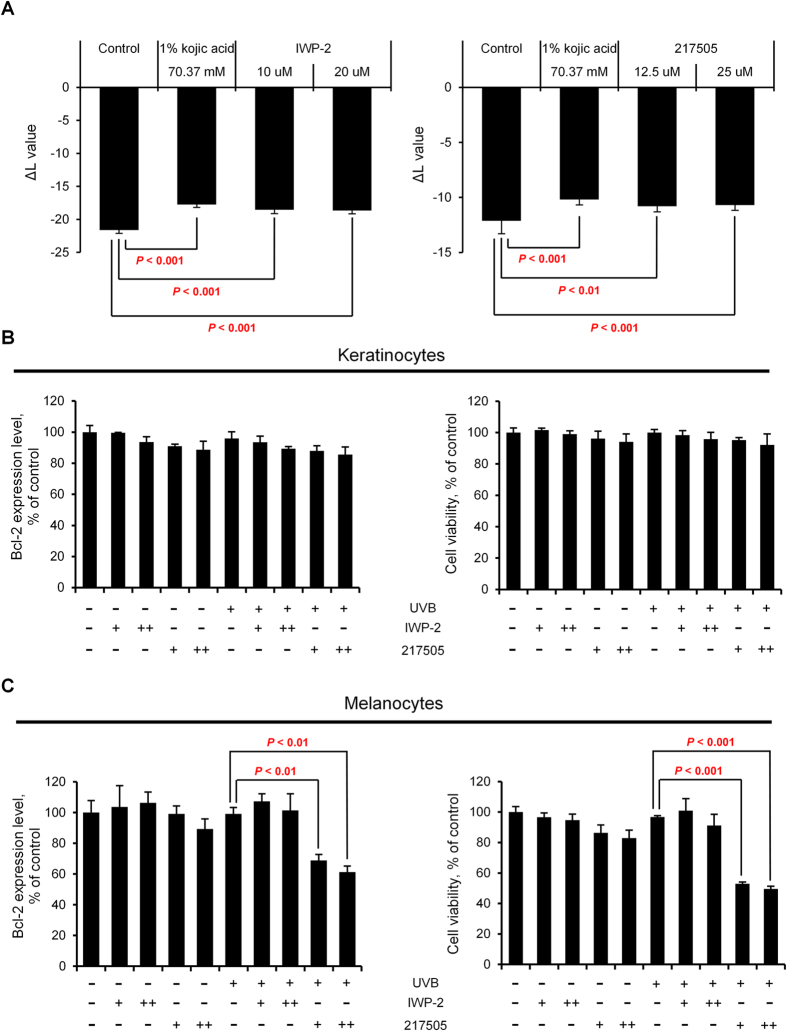
Effects of inhibition of beta-catenin or CREB on the UVB-induced skin pigmentation and Bcl-2 expression. (**A**) Graphs of the ΔL value of the UVB-irradiated skin equivalent model, MelanoDerms^TM^, exposed to the indicated concentrations of IWP-2 (an inhibitor of Wnt/beta-catenin pathway) or 217505 (**a** CBP-CREB interaction inhibitor). 1% kojic (70.37 mM) acid was used as a positive control. MelanoDerms^TM^ were grown at the air-liquid interface and the maintenance medium was replenished every 2 days. After a 9-day of exposure to the chemicals, pigmentation of the skin equivalent was assessed by comparing the change in L* value, a value of CIE 1976 (L*,a*,b*) color space representing the brightness. (**B** and **C**) Graphs of the Bcl-2 expression level and the cell viability in normal human keratinocytes and normal human melanocytes exposed to IWP-2 [+, 10 μM; ++, 20 μM] or 217505 [+, 12.5 μM; ++, 25 μM] with or without UVB irradiation. Cells were cultured in 6-well plate and exposed to UVB irradiation (10 mJ/cm^2^). The cells were then treated with IWP-2 or 217505 for 24 hours. After incubation, the cells were harvested and lysed. Bcl-2 expression level was measured by using human Bcl-2 ELISA kit (Abcam, Cambridge, U.K.). For the cell viability assay, the cells were treated with WST-1 (Roche Molecular Biochemicals, Mannheim, Germany). Cell viability was determined from absorbance (OD 450) measured by using microplate reader. Data represent the means + SD of three biological replicates. *P*-values were determined by Student’s *t* test; *P* < 0.05 was considered statistically significant. No statistically significant difference in the Bcl-2 expression level or the cell viability was observed between control and IWP-2 treated cells upon UVB irradiation (*P* > 0.05).

To validate the predictions made by the mathematical model, we used pharmacological inhibition of signaling proteins and monitored cellular responses to UVB. Pigmentation of the MelanoDerms, reconstituted human skin equivalents which have been applied to implement biological reactions that occur in physiological human skin such as keratinocytes-melanocytes interactions and skin pigmentation^49^, was assessed by measuring the ΔL value; higher ΔL values of the MelanoDerms indicate lighter colors as previously reported^50^. Note that the UVB dose of 10 mJ/cm^2^ was chosen for the biochemical validation of model simulation since exposing cells to the UVB irradiation at a dose higher than 10 mJ/cm^2^ significantly decreased the Bcl-2 expression level without any chemical treatment (Fig. S3). Exposing MelanoDerms to the chemical IWP-2 (an inhibitor of Wnt/beta-catenin pathway) or 217505 (a CBP-CREB interaction inhibitor) significantly increased the ΔL value when irradiated with 10 mJ/cm^2^ of UVB, indicating that inhibition of beta-catenin or CREB reduced the skin pigmentation (Fig. 4A). The kojic acid (70.37 mM), a proven skin depigmentation agent, was used as a positive control to confirm that the experimental conditions such as the reagents and equipment are functioning properly. We also examined the effects of the inhibition of beta-catenin or CREB on both the Bcl-2 expression and the survival rate of the cells. Note that in the model simulation, inhibition of either beta-catenin or CREB dramatically reduced melanin synthesis, but unlike beta-catenin, inhibition of CREB largely influenced the Bcl-2 expression. As a result, we found that the inhibition of beta-catenin by exposing keratinocytes or melanocytes to the chemical IWP-2 does not change the Bcl-2 expression level upon UVB irradiation (Fig. 4B, C). Furthermore, the results of the cell viability assay using WST-1 quantification showed that the viability of cells in response to UVB irradiation is not significantly affected by IWP-2 exposure (Fig. 4B, C). In contrast, the inhibition of CREB by exposing cells to the chemical 217505 has significantly decreased both the Bcl-2 expression level and the cell viability in melanocytes while not significantly changing those in keratinocytes upon UVB irradiation (Fig. 4B, C). Thus, these experimental data are consistent with the prediction from the mathematical simulation (Table 1) that the beta-catenin inhibition may be the most appropriate strategy for the reduction of UVB-induced melanin synthesis since it can effectively reduce the melanin synthesis while minimally affecting the apoptotic balance of the cells.

To further validate our model, we also compared the predicted effects of multiple compounds from the model simulation with the experimental effects of the same compounds (see Fig. S4 and Table S4 for details). As a result, we confirmed that the predictive value of our mathematical model is reasonably adequate for further investigation of an optimal target and its intervention strategy that can reduce the UVB-induced skin pigmentation.

## Discussion

Melanogenesis is a biological process that results in the synthesis of melanin which serves as the protective agent against UVB irradiation^51^. Diverse cellular responses, including cell survival and apoptosis, are affected during the melanogenesis through complicated regulatory mechanisms of feedback and crosstalk. Since perturbations in the apoptotic balance of the cells involved in the melanogenesis (keratinocytes and melanocytes) may result in various skin diseases^52^,^53^,^54^, research interest in the investigation of appropriate target strategies for the reduction of UVB-induced skin pigmentation has been increased. Considering the biological complexity of the underlying signaling network, however, there is a fundamental limitation in using only biological experimental techniques to investigate such safe and effective target strategies. To overcome the limitation, mathematical modeling and computer simulations have often been employed as useful tools, which facilitates the exploration of the hidden dynamics and underlying mechanisms of complex signal transduction systems^21^,^55^,^56^,^57^. In this study, we attempted to find an optimal target and its intervention strategy for the reduction of UVB-induced melanin synthesis using a systems biological approach by combining mathematical simulations and biochemical experiments. Our novel findings obtained from this interdisciplinary effort led to the following conclusion: inhibition of beta-catenin in the melanocytes effectively reduces melanin production while minimally influencing the Bcl-2 expression level.

Systems biology has emerged as a promising interdisciplinary field of biological research that aims to understand complex biological processes at a system level and to predict cellular behavior, which facilitates the drug development process^58^. The conventional process of drug discovery and development is time-consuming and costly, mainly due to the excessive efforts required for therapeutic target screening, animal model testing and clinical trial stages^59^. Systems biology can provide a set of qualified candidate target proteins by predicting the potential therapeutic effects of the drug treatment through *in silico* analysis, which may substantially shorten the time and cost of development while also improving the safety and efficacy of targeted therapies^59^. Meanwhile, various fields of industry confront similar challenging issues that medical and pharmacology research is currently facing. The safety assessment of the ingredients in cosmetic and hygienic products also involves heavy use of animal testing, which is time consuming, cost-ineffective, and most of all, causes unnecessary excessive sacrifice of the animal subjects. For this reason, the restriction in use of animal models for the commercial purposes has emerged; for instance, the European Union has prohibited the performance of animal testing for cosmetics^60^. Under these circumstances, there is a growing interest in developing non-animal alternative methods of toxicity testing and safety assessment such as mathematical modeling and computer simulations^60^. With these, the proposed systems biological approach might be an alternative way to effectively facilitate the product development process by identifying promising candidate cosmetic compounds based on model simulations of their potential effectiveness and toxicity prior to *in vivo* or *in vitro* testing.

In summary, from mathematical simulations combined with biochemical experiments, we found that the inhibition of beta-catenin can effectively reduce melanin production while minimally influencing the apoptotic balance of the cells. Our study provides a novel insight into the complex regulatory mechanism of melanogenesis and the identification of an optimal target and its intervention strategy for desired outcomes.

## Materials and Methods

### Reconstruction of the melanogenesis network

The melanogenesis network, consisting of 62 nodes and 113 directed links, was reconstructed by integrating all available fragmented information about individual proteins and their interactions through an extensive survey of the relevant literatures. The 113 links that comprise the melanogenesis network are described in Table S1: The ‘Source’ column refers to the molecules that regulate ‘Target’ molecules (e.g., enzymes, ligands); and the ‘Target’ column refers to the molecules being regulated by the ‘Source’ molecules (e.g., substrates, receptors). In the ‘Interaction’ column, ‘+’ and ‘-’ denote activation and inhibition, respectively. For the Boolean simulations, the activity state of each node except for the external-input node (UVB) was determined by its assigned logic table as described in Table S2. The logic tables were created upon the mechanistic information about the activation and/or inhibition of each signaling protein obtained from the relevant literatures (Table S2).

### Boolean simulation to measure the steady-state activity of each node

While the state space of the melanogenesis network is huge (2^62^ states), from numerous iterative simulations, we found that the state of the network appears to converge into a relatively small cycle within 200 time steps. Thus, to measure the steady-state activity of each node with various levels of input stimulations, we traced each Boolean function update for 1,000 time steps. For each simulation run, the input node is placed on a cycle that yields a desired percentage of ON, representing the intensity of input stimulation. For example, if the input is to be set at 50% ON, it would be put on a cycle of ‘101010’ or ‘010101’, which is held constant for the duration of the mathematical simulation. The intensity of the input ‘UVB’ was set to 0–100% ON with 1% interval. A small amount of background noise (2%, described below) was added to the input, which generates more biologically realistic behaviors. The average ‘1’s over the last 100 time steps were calculated and used for the steady-state activity of each node. Initial conditions of all nodes were randomized in every run (50% ‘0’, 50% ‘1’). The pigmentation state (pigmentation or depigmentation) and the apoptotic balance (cell survival or apoptosis) of the cells were determined by observing the steady-state activities of the nodes ‘Melanin’ and the ‘Bcl-2’, respectively.

### Adding noise to the input

Simulations were performed while giving a random noise to the input that forces it to vary chaotically within a range around the set input level. For example, if an input is set to 50% ON in a given run, 2% noise for the input would result in the input varying chaotically between 48% and 52% ON.

### Node control analysis

Node control analysis was performed by pinning the state of each internal regulatory node to either ‘0’ for inhibition or ‘1’ for constitutive activation. The perturbation effect of the node control on the three output nodes (melanin, Bcl-2 in keratinocytes, and Bcl-2 in melanocytes) in the malanogenesis network was measured. The perturbation effect 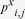 of an internal regulatory node *j* on an output node *i* for *x* perturbation (inhibition or constitutive activation) is defined as following equation 1:


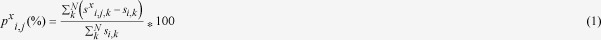


where 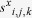 denotes the steady-state activity of the output node *i* with *x* perturbation of the internal regulatory node *j* for *k* level of UVB, 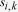 denotes the steady-state activity of output node *i* without any perturbation for *k* level of UVB, and *N* denotes the number of various levels of UVB in the range of 0%–100% with 1% interval.

### State-space analysis of the melanogenesis network

In the state-space analysis, the state of each node was updated based on its assigned logic table. For an external-input (UVB) stimulation, UVB was persistently applied by maintaining active (‘1’) state through the simulations. To identify the attractors, or the logical steady-states, Boolean simulations were performed until the state reached a fixed point or a cyclic attractor for a given initial network state. The basin size of an attractor, given in percentage, was estimated by calculating the proportion of the initial network states converging towards the attractor to the total number of randomly selected initial network states. The distribution of the estimated basin size was maintained even for smaller sampling sizes of initial network states (Fig. S1); therefore, 100,000 initial network states were used for each perturbation case. Note that attractors, in general, refer to the steady-states of a system in the absence of external-input energy (‘0’); hence, attractors under persistent UVB stimulation are in fact quasi-attractors.

### Reagents

Kojic acid was purchased from Sigma Chemical Co. (St. Louis, MO, USA). IWP-2 (a beta-catenin inhibitor) and salirasib (a Ras inhibitor) were obtained from Tocris Bioscience (Bristol, UK). 217505 (a CBP-CREB interaction inhibitor) was purchased from Calbiochem (Darmstadt, Germany). H-89 (a PKA inhibitor) was purchased from Sigma-Aldrich (St. Louis, USA).

### Pigmentation assessment in human skin equivalents

Dark (from African-American skin, MelanoDerms^TM^) human epidermal equivalents were purchased from MatTek Corp. (Ashland, MA, USA). MelanoDerms were grown at the air–liquid interface of the maintenance medium at 37 °C under 5% CO_2_ in a humidified incubator, and the medium was changed every 2 days. Each test sample was diluted in Dulbecco’s Phosphate Buffered Saline (DPBS) with concentrations which showed no cytotoxicity on skin equivalents, and treated on skin equivalents. DPBS and kojic acid (1%) were used for vehicle-treated and positive controls, respectively. The samples were treated with IWP-2, 217505, salirasib, or H-89 for 9 days on their surface. Pigmentation of the skin equivalents was assessed by comparing the change in L* value, a value of CIE 1976 (L*, a*, b*) color space representing the brightness. The level of pigmentation was monitored by calculating the difference (ΔL* value) between the mean L* values at day 9 and at day 0 for each skin equivalent. The mean and standard deviation of the L* values of the skin equivalents were assessed by measurement of L* values at 10 different, randomly chosen positions on each skin equivalent. The UVB dose of 10 mJ/cm^2^ was chosen for the biochemical experiments.

### Cell culture

The human epidermal melanocytes from the neonatal foreskin of darkly pigmented donors were purchased from Cascade Biologics (Portland, OR, USA) and cultured in Medium 254 (Cascade Biologics) supplemented with human melanocyte growth supplement (HMGS). Normal human melanocytes (NHMs) were confluently grown in Medium 254 and HMGS with at 37 °C and 5% CO_2_. The medium was changed every 2 days, and the cells were passaged upon reaching 80% confluence. For *in vitro* experiments, the cells were seeded into 12-well plates and incubated for 24 hours. The cells were then exposed to IWP-2 and 217505 for 24 hours, and harvested for the Bcl-2 ELISA assay. The UVB dose of 10 mJ/cm^2^ was chosen for the biochemical experiments.

### Bcl-2 ELISA assay

Bcl-2 was quantified by ELISA using Bcl-2 human ELISA kit (Abcam, Cambridge, MA, USA). Cell lysis, sample preparation and experimental procedure were carried out according to the manufacturer’s instruction. Briefly, the NHMs exposed to IWP-2 and 217505 were incubated in lysis buffer for 1 hour at room temperature, and centrifuged at 1000 x g for 15 minutes. The cleared lysates were harvested, and the samples and human Bcl-2 standard proteins were added in the microwell plate coated with monoclonal antibody to human Bcl-2. A biotin-conjugated anti-human Bcl-2 antibody was added in each well and incubated for 2 hours. After a wash step using wash buffer (PBS with 1% Tween 20), streptavidin-HRP was added and incubated for an hour. Following incubation, each well was washed for 3 times, and substrate solution (etramethyl-benzidine) was added to the wells. The reaction was terminated by addition of stop solution (1M Phosphoric acid), and absorbance at 450 nm of the samples was read on a SpectraMax 190 microplate reader (Molecular Devices Corp., Sunnyvale, CA, USA). The values were normalized based on the protein concentrations in each sample well.

## Author Contributions

K.-H.C. designed the project and supervised the research; H.-S.L., J.K., T.-J.C. and K.-H.C. performed the mathematical modeling and analysis; H.K.L. and Y.J.N. conceived and designed the experiments; M.-J.G. performed the biochemical experiments; and H.-S.L., M.-J.G., J.K., T.-J.C., Y.J.N. and K.-H.C. wrote the manuscript.

## Additional Information

**How to cite this article**: Lee, H.-S. *et al*. A systems-biological study on the identification of safe and effective molecular targets for the reduction of ultraviolet B-induced skin pigmentation. *Sci. Rep.* **5**, 10305; doi: 10.1038/srep10305 (2015).

## Supplementary Material

Supplementary Information

## Figures and Tables

**Table 1:** Potentially effective target strategies for the reduction of UVB-induced skin pigmentation.

Node	Perturbation	Δ Melanin (%)	Δ Bcl-2_K (%)	Δ Bcl-2_M (%)
b-catenin_M	Inhibition	−100.00	7.78	−8.15
MITFmRNA_M	Inhibition	−100.00	7.78	−23.75
MITFprotein_M	Inhibition	−100.00	7.78	−23.75
GSK3b_M	Constitutive activation	−100.00	7.78	−23.95
ERK_M	Inhibition	−100.00	7.78	−58.53
MEK_M	Inhibition	−100.00	7.78	−58.53
RAF_M	Inhibition	−100.00	7.78	−58.53
CREB_M	Inhibition	−100.00	7.78	−86.62
Akt_M	Inhibition	−100.00	7.78	−100.00
PDK1_M	Inhibition	−100.00	7.78	−100.00
PI3K_M	Inhibition	−100.00	7.78	−100.00
cAMP_M	Constitutive activation	−100.00	7.78	−100.00
MKK6_M	Constitutive activation	−100.00	7.78	−100.00
p38_M	Constitutive activation	−100.00	7.78	−100.00
ASK1_M	Constitutive activation	−100.00	7.78	−100.00

The list comprises node controls that significantly reduce (<–90%) the activity of node ‘melanin’ in response to UVB irradiation.
