# Supplementary material for: A systems-biological study on the identification of safe and effective molecular targets for the reduction of ultraviolet B-induced skin pigmentation
Source: Sci Rep. 2015 May 18;5:10305. doi: 10.1038/srep10305 (PMC4434836; doi:10.1038/srep10305)
Supplement: Supplementary Information [file srep10305-s1.pdf]

A systems biological study on the identification of safe and effective molecular targets for reduction of ultraviolet B-induced skin pigmentation

Ho-Sung Lee [1,2,*], Myeong-Jin Goh [3,*], Junil Kim [1,*], Taejun Choi [1], Hae Kwang Lee [3], Yong Joo Na [3,‡] and Kwang-Hyun Cho [1,2,†]

[1]Laboratory for Systems Biology and Bio-Inspired Engineering, Department of Bio and Brain Engineering, Korea Advanced Institute of Science and Technology, Daejeon, 305-701, Republic of Korea.

[2]Graduate School of Medical Science and Engineering, Korea Advanced Institute of Science and Technology, Daejeon, 305-701, Republic of Korea.

[3]Skin Research Institute, Amore Pacific R&D center, Gyeonggi-do, 446-729, Republic of Korea.

Supplementary Materials

[*] These authors contributed equally to this study.
[†]Corresponding author. E-mail: ckh@kaist.ac.kr, Phone: +82-42-350-4325, Fax: +82-42-350-4310, Web: http://sbie.kaist.ac.kr/.
[‡]Co-corresponding author. E-mail: nay@amorepacific.com

Table of Contents

I. Supplementary Figures

Supplementary Figure S1. The effect of UVA or UVB irradiation on the melanin content in human melanocytes.

Supplementary Figure S2. The estimated basin sizes for different sampling numbers of initial network states.

Supplementary Figure S3. The effect of UVB irradiation on the Bcl-2 expression level in human melanocytes.

Supplementary Figure S4. Effects of inhibition of beta-catenin, Ras, PKA, or a combination of Ras and PKA on the UVB-induced skin pigmentation.

II. Supplementary Tables

Supplementary Table S1. The 113 links comprising the melanogenesis network.

Supplementary Table S2. Logic tables for the Boolean network model.

Supplementary Table S3. *In silico* node control anaysis for the identification of appropriate strategies to reduce UVB-induced skin pigmentation.

Supplementary Table S4. *In silico* analysis of changes in UVB-induced melanin synthesis with respect to the inhibition of beta-catenin, Ras, PKA, or a combination of Ras and PKA.

III. Supplementary References

I. Supplementary Figures

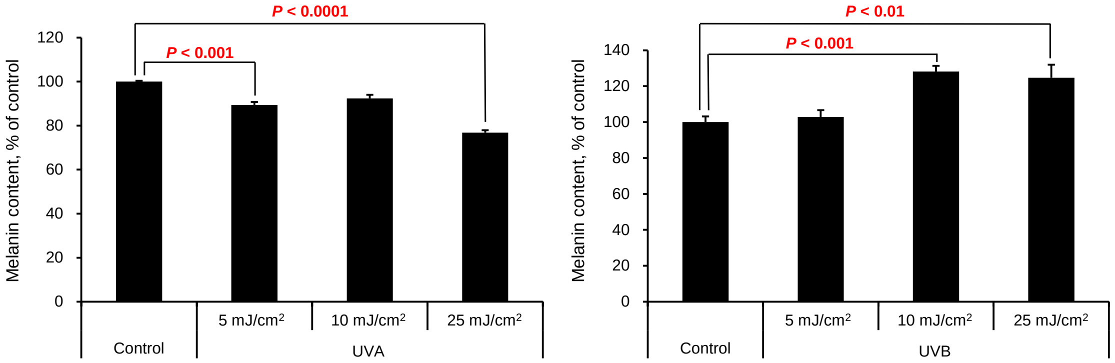

Supplementary Figure S1. The effect of UVA or UVB irradiation on the melanin content in human melanocytes.

Graphs of the melanin content in normal human melanocytes exposed to UVA or UVB irradiation at the indicated doses. Human melanocytes were cultured in 6-well plate and exposed to the indicated doses of UVA or UVB. After incubation, the melanocytes were harvested and dissolved in 1N NaOH solution. The melanin content was determined from absorbance (OD 475) measured by using microplate reader. The data represent the means + SD of three biological replicates. *P*-values were determined by Student's *t* test; $P < 0.05$ was considered statistically significant.

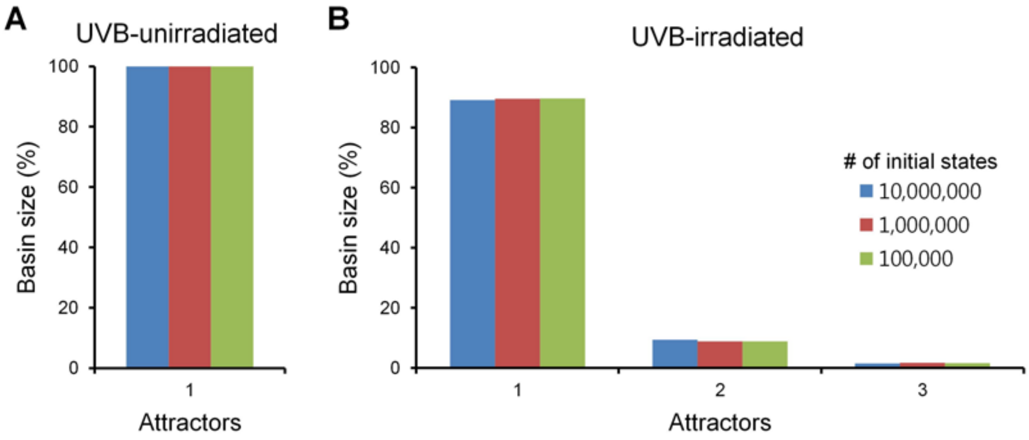

Supplementary Figure S2. The estimated basin sizes for different sampling numbers of initial network states.

The distributions of the estimated basin sizes are very similar regardless of the sampling number of initial states.

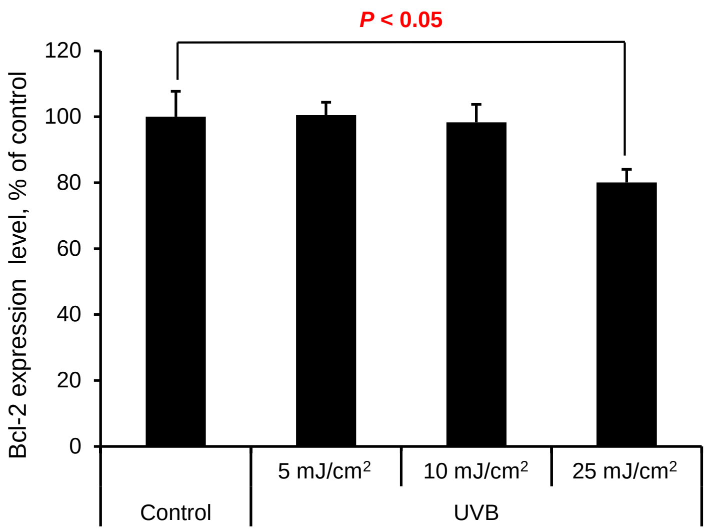

Supplementary Figure S3. The effect of UVB irradiation on the Bcl-2 expression level in human melanocytes.

Graphs of the Bcl-2 expression level in normal human melanocytes exposed to UVB irradiation at the indicated doses. Human melanocytes were cultured in 6-well plate and exposed to the indicated doses of UVB. After incubation, the melanocytes were harvested and lysed. Bcl-2 expression level was measured by using human Bcl-2 ELISA kit (Abcam, Cambridge, U.K.). The data represent the means + SD of three biological replicates. *P*-values were determined by Student's *t* test; $P < 0.05$ was considered statistically significant.

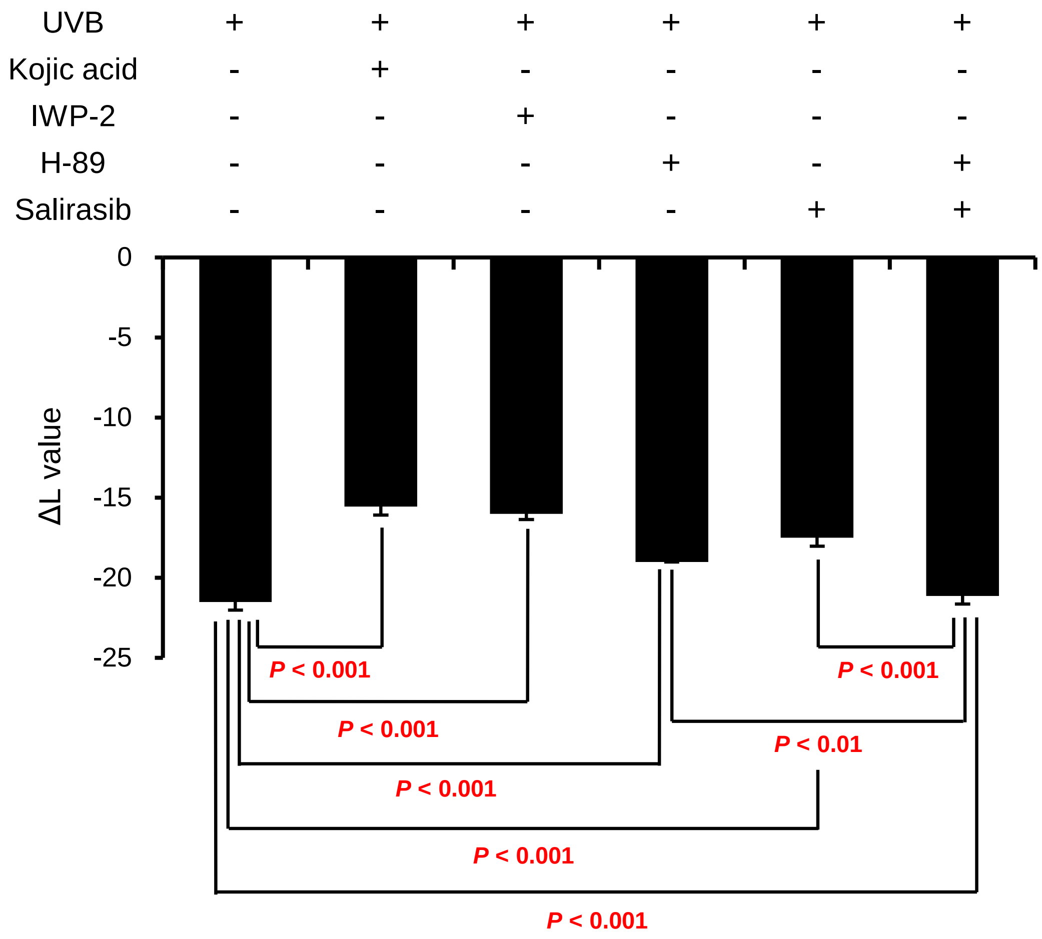

Supplementary Figure S4. Effects of inhibition of beta-catenin, Ras, PKA, or a combination of Ras and PKA on the UVB-induced skin pigmentation.

Graphs of the ΔL values of the MelanoDerms exposed to IWP-2 (a beta-catenin inhibitor, 20 μM), H-89 (a PKA inhibitor, 10 μM), salirasib (a Ras inhibitor, 50 μM), or a combined treatment with H-89 (10 μM) and salirasib (50 μM) upon UVB irradiation (10 mJ/cm^2). MelanoDerms exposed to a combined treatment with H-89 and salirasib showed the ΔL value lower than those treated with either H-89 or salirasib alone. 1% kojic (70.37 mM) acid was used as a positive control. MelanoDerms were grown at the air-liquid interface and the maintenance medium was replenished every 2 days. After a 9-day of exposure to the chemicals, pigmentation of the skin equivalents was assessed by comparing the change in L* value, a value of CIE 1976 (L*,a*,b*) color space representing the brightness. The data represent the means + SD of at least three biological replicates. *P*-values were determined by Student's *t* test; $P < 0.05$ was considered statistically significant. See Table S4 for the simulation results of changes in UVB-induced melanin synthesis with respect to the inhibition of beta-catenin, Ras, PKA, or a combination of Ras and PKA.

II. Supplementary Tables

Supplementary Table S1. The 113 links comprising the melanogenesis network.

Link index	Cellular type	Source	Interaction	Target	Reference
1	Extracellular Signal	UVB	+	ASK1	[1-3]
2	Extracellular Signal	UVB	+	EGFR	[1]
3	Extracellular Signal	UVB	+	IL-1	[4]
4	Extracellular Signal	UVB	-	PTEN	[1,5,6]
5	Keratinocytes	Melanin	-	IL-1	See the note[§].
6	Keratinocytes	ERK	-	PTEN	[6]
7	Keratinocytes	Akt	-	PTEN	[6]
8	Keratinocytes	Melanin	+	PTEN	See the note[§].
9	Keratinocytes	Melanin	-	EGFR	See the note[§].
10	Keratinocytes	EGFR	+	PI3K	[7]
11	Keratinocytes	Akt	-	ASK1	[8]
12	Keratinocytes	Melanin	-	ASK1	See the note[§].
13	Keratinocytes	EGFR	+	SG	[9-11]
14	Keratinocytes	ERK	-	SG	[9,12]
15	Keratinocytes	PI3K	+	PDK1	[13,14]
16	Keratinocytes	PTEN	-	PDK1	[15]
17	Keratinocytes	ASK1	+	MKK6	[16]
18	Keratinocytes	Akt	-	MKK4	[17]
19	Keratinocytes	ASK1	+	MKK4	[16]
20	Keratinocytes	SG	+	Ras	[18]
21	Keratinocytes	PDK1	+	Akt	[14,19]
22	Keratinocytes	MKK6	+	p38	[20]
23	Keratinocytes	MKK4	+	JNK	[20]
24	Keratinocytes	Akt	-	Raf	[21]
25	Keratinocytes	Ras	+	Raf	[22]
26	Keratinocytes	Akt	-	GSK3b	[23]
27	Keratinocytes	GSK3b	-	b-catenin	[24]
28	Keratinocytes	Raf	+	MEK	[25-27]
29	Keratinocytes	Akt	+	MDM2	[28]
30	Keratinocytes	GSK3b	-	NFAT	[29,30]
31	Keratinocytes	b-catenin	+	COX-2	[31]
32	Keratinocytes	GSK3b	-	COX-2	[32]
33	Keratinocytes	NFAT	+	COX-2	[33]
34	Keratinocytes	p38	+	COX-2	[34]
35	Keratinocytes	MEK	+	ERK	[35,36]
36	Keratinocytes	p38	-	ERK	[37,38]
37	Keratinocytes	ERK	+	p53	[39]

38	Keratinocytes	MDM2	-	p53	40
39	Keratinocytes	p38	+	p53	41
40	Keratinocytes	ERK	+	RSK	42,43
41	Keratinocytes	Akt	+	Bcl-2	44,45
42	Keratinocytes	ERK	+	Bcl-2	46
43	Keratinocytes	JNK	-	Bcl-2	47,48
44	Keratinocytes	b-catenin	+	Bcl-2	49
45	Keratinocytes	GSK3b	+	Bcl-2	50,51
46	Keratinocytes	p38	-	Bcl-2	52
47	Keratinocytes	p53	-	Bcl-2	53-55
48	Keratinocytes	RSK	+	Bcl-2	43,56,57
49	Keratinocytes	IL-1	+	ET-1	58
50	Keratinocytes	p53	+	ET-1	59,60
51	Keratinocytes	IL-1	+	SCF	61
52	Keratinocytes	p53	+	SCF	59
53	Keratinocytes	p53	+	a-MSH	59
54	Keratinocytes	COX-2	+	PGE2	62,63
55	Keratinocytes	IL-1	+	PGE2	62,63
56	Paracrine	ET-1	+	ETR	64
57	Paracrine	SCF	+	c-Kit	65,66
58	Paracrine	a-MSH	+	MC1R	67
59	Paracrine	PGE2	+	EP4	68
60	Melanocytes	ETR	+	PKC	64
61	Melanocytes	c-Kit	+	SG	69
62	Melanocytes	ERK	-	SG	9,12
63	Melanocytes	MC1R	+	AC	70,71
64	Melanocytes	Akt	-	ASK1	8
65	Melanocytes	PKC	+	PI3K	72
66	Melanocytes	Ras	+	PI3K	73
67	Melanocytes	cAMP	-	PI3K	74
68	Melanocytes	SG	+	Ras	18
69	Melanocytes	cAMP	+	Ras	75
70	Melanocytes	EP4	+	cAMP	68
71	Melanocytes	AC	+	cAMP	76
72	Melanocytes	PDE	-	cAMP	77,78
73	Melanocytes	ASK1	+	MKK4	16
74	Melanocytes	Akt	-	MKK4	17
75	Melanocytes	PI3K	+	PDK1	13,14
76	Melanocytes	PKC	+	Raf	79
77	Melanocytes	Ras	+	Raf	22,26
78	Melanocytes	cAMP	+	PKA	76

79	Melanocytes	ASK1	+	MKK6	[16]
80	Melanocytes	PDK1	+	Akt	[14,19]
81	Melanocytes	Raf	+	MEK	[25-27]
82	Melanocytes	PKA	+	PDE	[80,81]
83	Melanocytes	MKK6	+	p38	[20]
84	Melanocytes	Akt	-	GSK3b	[23]
85	Melanocytes	MEK	+	ERK	[35,36]
86	Melanocytes	p38	-	ERK	[37]
87	Melanocytes	p38	+	MSK	[43,82]
88	Melanocytes	ERK	+	MSK	[43,82]
89	Melanocytes	MKK4	+	JNK	[20]
90	Melanocytes	Akt	+	MDM2	[28]
91	Melanocytes	ERK	+	RSK	[42,43]
92	Melanocytes	p38	+	p53	[41]
93	Melanocytes	MDM2	-	p53	[40]
94	Melanocytes	MITFprotein	-	p53	[83]
95	Melanocytes	GSK3b	-	b-catenin	[24]
96	Melanocytes	PKA	+	CREB	[76]
97	Melanocytes	Akt	+	CREB	[45,84]
98	Melanocytes	MSK	+	CREB	[43]
99	Melanocytes	Akt	+	Bcl-2	[44,45]
100	Melanocytes	p38	-	Bcl-2	[52]
101	Melanocytes	ERK	+	Bcl-2	[46]
102	Melanocytes	JNK	-	Bcl-2	[47,48]
103	Melanocytes	RSK	+	Bcl-2	[43,56,57]
104	Keratinocytes	b-catenin	+	Bcl-2	[49]
105	Keratinocytes	GSK3b	+	Bcl-2	[50,51]
106	Melanocytes	p53	-	Bcl-2	[53-55]
107	Melanocytes	CREB	+	Bcl-2	[45,85]
108	Melanocytes	MITFprotein	+	Bcl-2	[86]
109	Melanocytes	b-catenin	+	MITFmRNA	[87]
110	Melanocytes	CREB	+	MITFmRNA	[88]
111	Melanocytes	ERK	+	MITFprotein	[89-91]
112	Melanocytes	MITFmRNA	+	MITFprotein	[92]
113	Melanocytes	MITFprotein	+	Melanin	[92]

[§]Links 5, 8, 9, and 12 were included to represent the photoprotective role of epidermal melanin against UVB irradiation.

IL-1, interleukin 1; PTEN, phosphatase and tensin homolog; EGFR, epidermal growth factor receptor; PI3K, phosphatidylinositol 3-kinase; ASK1, apoptosis signal-regulating kinase 1; SG, the growth factor receptor–bound protein 2 (Grb2) and Son of Sevenless (SOS) complex; PDK1,

phosphoinositide-dependent kinase 1; MKK6, mitogen-activated protein kinase (MAPK) 6; MKK4, mitogen-activated protein kinase (MAPK) 4; JNK, c-Jun N-terminal kinase; GSK3b, Glycogen synthase kinase-3 beta; b-catenin, beta-catenin; ERK, extracellular signal-regulated kinase; MEK, MAPK/ERK kinase; MDM2, mouse double minute 2 homolog; NFAT, nuclear factor of activated T-cells; COX-2, cyclooxygenase (COX-2); RSK, ribosomal s6 kinase; ETR, endothelin receptor; MC1R, melanocortin 1 receptor; EP4, prostaglandin E receptor 4; PKC, protein kinase C; AC, adenylyl cyclase; cAMP, cyclic adenosine monophosphate; PKA, protein kinas A; PDE, phosphodiesterase; MSK, mitogen- and stress-activated kinase and CREB, cAMP response element-binding protein.

Supplementary Table S2. Logic tables of the Boolean network model.

<table>
<tr><th>Node index</th><th>Logic table</th><th>Remarks</th></tr>
<tr><td>1</td><td>
<table>
<tr><th>Melanin</th><th>UVB</th><th>IL-1_K</th></tr>
<tr><td>0</td><td>0</td><td>0</td></tr>
<tr><td>0</td><td>1</td><td>1</td></tr>
<tr><td>1</td><td>0</td><td>0</td></tr>
<tr><td>1</td><td>1</td><td>0</td></tr>
</table>
</td><td>IL-1 activation in human keratinocytes is augmented by UVB irradiation.</td></tr>
<tr><td>2</td><td>
<table>
<tr><th>Melanin</th><th>UVB</th><th>Akt_K</th><th>ERK_K</th><th>PTEN_K</th></tr>
<tr><td>0</td><td>0</td><td>0</td><td>0</td><td>1</td></tr>
<tr><td>0</td><td>0</td><td>0</td><td>1</td><td>0</td></tr>
<tr><td>0</td><td>0</td><td>1</td><td>0</td><td>0</td></tr>
<tr><td>0</td><td>0</td><td>1</td><td>1</td><td>0</td></tr>
<tr><td>0</td><td>1</td><td>0</td><td>0</td><td>0</td></tr>
<tr><td>0</td><td>1</td><td>0</td><td>1</td><td>0</td></tr>
<tr><td>0</td><td>1</td><td>1</td><td>0</td><td>0</td></tr>
<tr><td>0</td><td>1</td><td>1</td><td>1</td><td>0</td></tr>
<tr><td>1</td><td>0</td><td>0</td><td>0</td><td>1</td></tr>
<tr><td>1</td><td>0</td><td>0</td><td>1</td><td>0</td></tr>
<tr><td>1</td><td>0</td><td>1</td><td>0</td><td>0</td></tr>
<tr><td>1</td><td>0</td><td>1</td><td>1</td><td>0</td></tr>
<tr><td>1</td><td>1</td><td>0</td><td>0</td><td>1</td></tr>
<tr><td>1</td><td>1</td><td>0</td><td>1</td><td>0</td></tr>
<tr><td>1</td><td>1</td><td>1</td><td>0</td><td>0</td></tr>
<tr><td>1</td><td>1</td><td>1</td><td>1</td><td>0</td></tr>
</table>
</td><td>UVB irradiation inhibits PTEN function by promoting its phosphorylation.
Phosphorylation of PTEN downregulates its lipid phosphatase function and protein stability.
UVB induced ERK/AKT-dependent PTEN suppression promotes survival of epidermal keratinocytes.</td></tr>
<tr><td>3</td><td>
<table>
<tr><th>Melanin</th><th>UVB</th><th>EGFR_K</th></tr>
<tr><td>0</td><td>0</td><td>0</td></tr>
<tr><td>0</td><td>1</td><td>1</td></tr>
<tr><td>1</td><td>0</td><td>0</td></tr>
<tr><td>1</td><td>1</td><td>0</td></tr>
</table>
</td><td>UVB irradiation induces phosphorylation of EGFR and increases its kinase activity.</td></tr>
<tr><td>4</td><td>
<table>
<tr><th>EGFR_K</th><th>PI3K_K</th></tr>
<tr><td>0</td><td>0</td></tr>
<tr><td>1</td><td>1</td></tr>
</table>
</td><td>EGFR-mediated phosphorylation of Gab1 results in PI3K activation.</td></tr>
</table>

5

Melanin	UVB	Akt_K	ASK1_K
0	0	0	0
0	0	1	0
0	1	0	1
0	1	1	0
1	0	0	0
1	0	1	0
1	1	0	0
1	1	1	0

UVB irradiation induces ASK1 activation.
Akt phosphorylates and negatively regulates ASK1 activity.

6

ERK_K	EGFR_K	SG_K
0	0	0
0	1	1
1	0	0
1	1	0

EGFR interacts with and activates SG complex, which in turn activates downstream Ras.
Activated ERK phosphorylates Sos and promotes disassociation of the SG complex.

7

PTEN_K	PI3K_K	PDK1_K
0	0	0
0	1	1
1	0	0
1	1	0

PI3K promotes the binding of PDK1 with PIP3, which in turn activates the downstream Akt kinase.
PTEN acts as a phosphatase to dephosphorylate PIP3, resulting in the deactivation of PDK1.

8

ASK1_K	MKK6_K
0	0
1	1

ASK1 phosphorylates and activates MKK6.

9

ASK1_K	Akt_K	MKK4_K
0	0	0
0	1	0
1	0	1
1	1	0

ASK1 activates MKK4 upon UVB irradiation.
Akt negatively regulates MKK4 activity by means of phosphorylation.

10

SG_K	Ras_K
0	0
1	1

SG complex catalyzes the exchange of GDP with GTP and induces Ras activation.

11

PDK1_K	Akt_K
0	0
1	1

Upon UVB irradiation, PDK1 phosphorylates Akt to promote its activation.

12

MKK6_K	p38_K
0	0
1	1

UVB-induced active MKK6 phosphorylates and activates p38.

13

MKK4_K	JNK_K
0	0
1	1

Active MKK4 directly phosphorylates JNK and promotes its activation.

14

Ras_K	Akt_K	Raf_K
0	0	0
0	1	0
1	0	1
1	1	0

Ras directly interacts with and activates Raf.
Akt phosphorylates and inhibits Ser/Thr kinase Raf.

15

Akt_K	GSK3b_K
0	1
1	0

AKT phosphorylates and inhibits kinase activity of GSK3b.

16

GSK3b_K	b-catenin_K
0	1
1	0

GSK3b phosphorylates b-catenin and promotes its degradation.

17

Raf_K	MEK_K
0	0
1	1

Raf phosphorylates and promotes MEK activation.

18

Akt_K	MDM2_K
0	0
1	1

Akt promotes nuclear localization and activation of MDM2 by means of phosphorylation.

19

GSK3b_K	NFAT_K
0	1
1	0

NFAT phosphorylation by active GSK3b suppresses its nuclear translocation, which reduces its DNA binding activity.

20

b-catenin_K	GSK3b_K	p38_K	NFAT_K	COX-2_K
0	0	0	0	0
0	0	0	1	1
0	0	1	0	1
0	0	1	1	1
0	1	0	0	0
0	1	0	1	0
0	1	1	0	0
0	1	1	1	0
1	0	0	0	1
1	0	0	1	1
1	0	1	0	1
1	0	1	1	1
1	1	0	0	0
1	1	0	1	0
1	1	1	0	0
1	1	1	1	0

p38, NFAT, or b-catenin is needed for the transcriptional activation of COX-2, hence forming an OR relation.
Inhibition of COX-2 by GSK3b is dominant to the positive regulators.

21

MEK_K	p38_K	ERK_K
0	0	0
0	1	0
1	0	1
1	1	0

MEK phosphorylates and activates ERK.
When both MEK and p38 are activated, dephosphorylation of ERK occurs.

22

MDM2_K	ERK_K	p38_K	p53_K
0	0	0	0
0	0	1	1
0	1	0	1
0	1	1	1
1	0	0	0
1	0	1	0
1	1	0	0
1	1	1	0

ERK or p38 kinase phosphorylates p53 protein in response to UVB irradiation, leading to its activation.
The nuclear localization of p53, which is necessary for its transcriptional activation, is inhibited by direct binding of p53 to MDM2.

23

ERK_K	RSK_K
0	0
1	1

RSK is directly phosphorylated by ERK, which promotes its kinase activity.

	JNK_K	p53_K	p38_K	b-catenin_K	GSK3b_K	Akt_K	ERK_K	RSK_K	Bcl-2_K
	0	0	0	0	0	0	0	0	1
	0	0	0	0	0	0	0	1	1
	0	0	0	0	0	0	1	0	1
	0	0	0	0	0	0	1	1	1
	0	0	0	0	0	1	0	0	1
	0	0	0	0	0	1	0	1	1
	0	0	0	0	0	1	1	0	1
	0	0	0	0	0	1	1	1	1
	0	0	0	0	1	0	0	0	1
	0	0	0	0	1	0	0	1	1
	0	0	0	0	1	0	1	0	1
	0	0	0	0	1	0	1	1	1
	0	0	0	0	1	1	0	0	1
	0	0	0	0	1	1	0	1	1
	0	0	0	0	1	1	1	0	1
	0	0	0	0	1	1	1	1	1
	0	0	0	1	0	0	0	0	1
	0	0	0	1	0	0	0	1	1
	0	0	0	1	0	0	1	0	1
	0	0	0	1	0	0	1	1	1
	0	0	0	1	0	1	0	0	1
24	0	0	0	1	0	1	0	1	1
	0	0	0	1	0	1	1	0	1
	0	0	0	1	0	1	1	1	1
	0	0	0	1	1	0	0	0	1
	0	0	0	1	1	0	0	1	1
	0	0	0	1	1	0	1	0	1
	0	0	0	1	1	0	1	1	1
	0	0	0	1	1	1	0	0	1
	0	0	0	1	1	1	0	1	1
	0	0	0	1	1	1	1	0	1
	0	0	0	1	1	1	1	1	1
	0	0	1	0	0	0	0	0	0
	0	0	1	0	0	0	0	1	0
	0	0	1	0	0	0	1	0	0
	0	0	1	0	0	0	1	1	0
	0	0	1	0	0	1	0	0	0
	0	0	1	0	0	1	0	1	0
	0	0	1	0	0	1	1	0	0
	0	0	1	0	0	1	1	1	0
	0	0	1	0	1	0	0	0	0
	0	0	1	0	1	0	0	1	0
	0	0	1	0	1	0	1	0	0
	0	0	1	0	1	0	1	1	0

Phosphorylation of BAD (a Bcl-2 antagonist) by Akt, ERK, or RSK is needed for the full activation of Bcl-2 protein.

Beta-catenin and GSK3-beta upregulates Bcl-2 expression level.

The activity of Bcl-2 is suppressed if any of its negative regulators is activated.

Hence, the activation condition for Bcl-2_M is 'AND(OR(Akt_K,ERK_K,RSK_K , b-catenin_K, GSK3b_K), NOT(OR(p53_K, JNK_K, p38_K)))'.

	0	0	1	0	1	1	0	1	0	
	0	0	1	0	1	1	1	0	0	
	0	0	1	0	1	1	1	1	0	
	0	0	1	1	0	0	0	0	0	
	0	0	1	1	0	0	0	1	0	
	0	0	1	1	0	0	1	0	0	
	0	0	1	1	0	0	1	1	0	
	0	0	1	1	0	1	0	0	0	
	0	0	1	1	0	1	0	1	0	
	0	0	1	1	0	1	1	0	0	
	0	0	1	1	0	1	1	1	0	
	0	0	1	1	1	0	0	0	0	
	0	0	1	1	1	0	0	1	0	
	0	0	1	1	1	0	1	0	0	
	0	0	1	1	1	0	1	1	0	
	0	0	1	1	1	1	0	0	0	
	0	0	1	1	1	1	0	1	0	
	0	0	1	1	1	1	1	0	0	
	0	0	1	1	1	1	1	1	0	
	0	1	0	0	0	0	0	0	0	
	0	1	0	0	0	0	0	1	1	
	0	1	0	0	0	0	1	0	1	
	0	1	0	0	0	0	1	1	0	
	0	1	0	0	0	1	0	0	1	
24	0	1	0	0	0	1	0	1	0	Phosphorylation of BAD (a Bcl-2 antagonist) by Akt, ERK, or RSK is needed for the full activation of Bcl-2 protein.<br>Beta-catenin and GSK3-beta upregulates Bcl-2 expression level.<br>The activity of Bcl-2 is suppressed if any of its negative regulators is activated.<br>Hence, the activation condition for Bcl-2_M is 'AND(OR(Akt_K,ERK_K,RSK_K, b-catenin_K, GSK3b_K), NOT(OR(p53_K, JNK_K, p38_K)))'.
	0	1	0	0	0	1	1	0	0	
	0	1	0	0	0	1	1	1	0	
	0	1	0	0	1	0	0	0	0	
	0	1	0	0	1	0	0	1	1	
	0	1	0	0	1	0	1	0	1	
	0	1	0	0	1	0	1	1	0	
	0	1	0	0	1	1	0	0	1	
	0	1	0	0	1	1	0	1	0	
	0	1	0	0	1	1	1	0	0	
	0	1	0	0	1	1	1	1	0	
	0	1	0	1	0	0	0	0	0	
	0	1	0	1	0	0	0	1	1	
	0	1	0	1	0	0	1	0	1	
	0	1	0	1	0	0	1	1	0	
	0	1	0	1	0	1	0	0	1	
	0	1	0	1	0	1	0	1	0	
	0	1	0	1	0	1	1	0	0	
	0	1	0	1	0	1	1	1	0	
	0	1	0	1	1	0	0	0	0	
	0	1	0	1	1	0	0	1	1	
	0	1	0	1	1	0	1	0	1	
	0	1	0	1	1	0	1	1	0	
	0	1	0	1	1	1	0	0	1	
	0	1	0	1	1	1	0	1	0	

	0	1	0	1	1	1	1	0	0	
	0	1	0	1	1	1	1	1	0	
	0	1	1	0	0	0	0	0	0	
	0	1	1	0	0	0	0	1	0	
	0	1	1	0	0	0	1	0	0	
	0	1	1	0	0	0	1	1	0	
	0	1	1	0	0	1	0	0	0	
	0	1	1	0	0	1	0	1	0	
	0	1	1	0	0	1	1	0	0	
	0	1	1	0	0	1	1	1	0	
	0	1	1	0	1	0	0	0	0	
	0	1	1	0	1	0	0	1	0	
	0	1	1	0	1	0	1	0	0	
	0	1	1	0	1	0	1	1	0	
	0	1	1	0	1	1	0	0	0	
	0	1	1	0	1	1	0	1	0	
	0	1	1	0	1	1	1	0	0	
	0	1	1	0	1	1	1	1	0	
	0	1	1	1	0	0	0	0	0	
	0	1	1	1	0	0	0	1	0	
	0	1	1	1	0	0	1	0	0	
	0	1	1	1	0	0	1	1	0	
	0	1	1	1	0	1	0	0	0	
	0	1	1	1	0	1	0	1	0	
24	0	1	1	1	0	1	1	0	0	Phosphorylation of BAD (a Bcl-2 antagonist) by Akt, ERK, or RSK is needed for the full activation of Bcl-2 protein.<br>Beta-catenin and GSK3-beta upregulates Bcl-2 expression level.<br>The activity of Bcl-2 is suppressed if any of its negative regulators is activated.<br>Hence, the activation condition for Bcl-2_M is 'AND(OR(Akt_K, ERK_K, RSK_K, b-catenin_K, GSK3b_K), NOT(OR(p53_K, JNK_K, p38_K)))'.
	0	1	1	1	0	1	1	1	0	
	0	1	1	1	1	0	0	0	0	
	0	1	1	1	1	0	0	1	0	
	0	1	1	1	1	0	1	0	0	
	0	1	1	1	1	0	1	1	0	
	0	1	1	1	1	1	0	0	0	
	0	1	1	1	1	1	0	1	0	
	0	1	1	1	1	1	1	0	0	
	0	1	1	1	1	1	1	1	0	
	1	0	0	0	0	0	0	0	0	
	1	0	0	0	0	0	0	1	1	
	1	0	0	0	0	0	1	0	1	
	1	0	0	0	0	0	1	1	0	
	1	0	0	0	0	1	0	0	1	
	1	0	0	0	0	1	0	1	0	
	1	0	0	0	0	1	1	0	0	
	1	0	0	0	0	1	1	1	0	
	1	0	0	0	1	0	0	0	0	
	1	0	0	0	1	0	0	1	1	
	1	0	0	0	1	0	1	0	1	
	1	0	0	0	1	0	1	1	0	
	1	0	0	0	1	1	0	0	1	
	1	0	0	0	1	1	0	1	0	
	1	0	0	0	1	1	1	0	0	

	1	0	0	0	1	1	1	1	0	
	1	0	0	1	0	0	0	0	0	
	1	0	0	1	0	0	0	1	1	
	1	0	0	1	0	0	1	0	1	
	1	0	0	1	0	0	1	1	0	
	1	0	0	1	0	1	0	0	1	
	1	0	0	1	0	1	0	1	0	
	1	0	0	1	0	1	1	0	0	
	1	0	0	1	0	1	1	1	0	
	1	0	0	1	1	0	0	0	0	
	1	0	0	1	1	0	0	1	1	
	1	0	0	1	1	0	1	0	1	
	1	0	0	1	1	0	1	1	0	
	1	0	0	1	1	1	0	0	1	
	1	0	0	1	1	1	0	1	0	
	1	0	0	1	1	1	1	0	0	
	1	0	0	1	1	1	1	1	0	
	1	0	1	0	0	0	0	0	0	
	1	0	1	0	0	0	0	1	0	
	1	0	1	0	0	0	1	0	0	
	1	0	1	0	0	0	1	1	0	
	1	0	1	0	0	1	0	0	0	
	1	0	1	0	0	1	0	1	0	
	1	0	1	0	0	1	1	0	0	
24	1	0	1	0	0	1	1	1	0	Phosphorylation of BAD (a Bcl-2 antagonist) by Akt, ERK, or RSK is needed for the full activation of Bcl-2 protein.<br>Beta-catenin and GSK3-beta upregulates Bcl-2 expression level.<br>The activity of Bcl-2 is suppressed if any of its negative regulators is activated.<br>Hence, the activation condition for Bcl-2_M is 'AND(OR(Akt_K, ERK_K, RSK_K, b-catenin_K, GSK3b_K), NOT(OR(p53_K, JNK_K, p38_K)))'.
	1	0	1	0	1	0	0	0	0	
	1	0	1	0	1	0	0	1	0	
	1	0	1	0	1	0	1	0	0	
	1	0	1	0	1	0	1	1	0	
	1	0	1	0	1	1	0	0	0	
	1	0	1	0	1	1	0	1	0	
	1	0	1	0	1	1	1	0	0	
	1	0	1	0	1	1	1	1	0	
	1	0	1	1	0	0	0	0	0	
	1	0	1	1	0	0	0	1	0	
	1	0	1	1	0	0	1	0	0	
	1	0	1	1	0	0	1	1	0	
	1	0	1	1	0	1	0	0	0	
	1	0	1	1	0	1	0	1	0	
	1	0	1	1	0	1	1	0	0	
	1	0	1	1	0	1	1	1	0	
	1	0	1	1	1	0	0	0	0	
	1	0	1	1	1	0	0	1	0	
	1	0	1	1	1	0	1	0	0	
	1	0	1	1	1	0	1	1	0	
	1	0	1	1	1	1	0	0	0	
	1	0	1	1	1	1	0	1	0	
	1	0	1	1	1	1	1	0	0	
	1	0	1	1	1	1	1	1	0	

	1	1	0	0	0	0	0	0	0	
	1	1	0	0	0	0	0	1	0	
	1	1	0	0	0	0	1	0	0	
	1	1	0	0	0	0	1	1	0	
	1	1	0	0	0	1	0	0	0	
	1	1	0	0	0	1	0	1	0	
	1	1	0	0	0	1	1	0	0	
	1	1	0	0	0	1	1	1	0	
	1	1	0	0	1	0	0	0	0	
	1	1	0	0	1	0	0	1	0	
	1	1	0	0	1	0	1	0	0	
	1	1	0	0	1	0	1	1	0	
	1	1	0	0	1	1	0	0	0	
	1	1	0	0	1	1	0	1	0	
	1	1	0	0	1	1	1	0	0	
	1	1	0	0	1	1	1	1	0	Phosphorylation of BAD (a Bcl-2 antagonist) by Akt, ERK, or RSK is needed for the full activation of Bcl-2 protein.<br>Beta-catenin and GSK3-beta upregulates Bcl-2 expression level.<br>The activity of Bcl-2 is suppressed if any of its negative regulators is activated.<br>Hence, the activation condition for Bcl-2_M is 'AND(OR(Akt_K, ERK_K, RSK_K, b-catenin_K, GSK3b_K), NOT(OR(p53_K, JNK_K, p38_K)))'.
	1	1	0	1	0	0	0	0	0	
	1	1	0	1	0	0	0	1	0	
	1	1	0	1	0	0	1	0	0	
	1	1	0	1	0	0	1	1	0	
	1	1	0	1	0	1	0	0	0	
	1	1	0	1	0	1	0	1	0	
	1	1	0	1	0	1	1	0	0	
	1	1	0	1	0	1	1	1	0	
24	1	1	0	1	1	0	0	0	0	
	1	1	0	1	1	0	0	1	0	
	1	1	0	1	1	0	1	0	0	
	1	1	0	1	1	0	1	1	0	
	1	1	0	1	1	1	0	0	0	
	1	1	0	1	1	1	0	1	0	
	1	1	0	1	1	1	1	0	0	
	1	1	0	1	1	1	1	1	0	
	1	1	1	0	0	0	0	0	0	
	1	1	1	0	0	0	0	1	0	
	1	1	1	0	0	0	1	0	0	
	1	1	1	0	0	0	1	1	0	
	1	1	1	0	0	1	0	0	0	
	1	1	1	0	0	1	0	1	0	
	1	1	1	0	0	1	1	0	0	
	1	1	1	0	0	1	1	1	0	
	1	1	1	0	1	0	0	0	0	
	1	1	1	0	1	0	0	1	0	
	1	1	1	0	1	0	1	0	0	
	1	1	1	0	1	0	1	1	0	
	1	1	1	0	1	1	0	0	0	
	1	1	1	0	1	1	0	1	0	
	1	1	1	0	1	1	1	0	0	
	1	1	1	0	1	1	1	1	0	
	1	1	1	1	0	0	0	0	0	

24

1	1	1	1	0	0	0	1	0
1	1	1	1	0	0	1	0	0
1	1	1	1	0	0	1	1	0
1	1	1	1	0	1	0	0	0
1	1	1	1	0	1	0	1	0
1	1	1	1	0	1	1	0	0
1	1	1	1	0	1	1	1	0
1	1	1	1	1	0	0	0	0
1	1	1	1	1	0	0	1	0
1	1	1	1	1	0	1	0	0
1	1	1	1	1	0	1	1	0
1	1	1	1	1	1	0	0	0
1	1	1	1	1	1	0	1	0
1	1	1	1	1	1	1	0	0
1	1	1	1	1	1	1	1	0

Phosphorylation of BAD (a Bcl-2 antagonist) by Akt, ERK, or RSK is needed for the full activation of Bcl-2 protein.
Beta-catenin and GSK3-beta upregulates Bcl-2 expression level.
The activity of Bcl-2 is suppressed if any of its negative regulators is activated.
Hence, the activation condition for Bcl-2_M is 'AND(OR(Akt_K, ERK_K, RSK_K, b-catenin_K, GSK3b_K), NOT(OR(p53_K, JNK_K, p38_K)))'.

25

IL-1_K	p53_K	ET-1_K
0	0	0
0	1	1
1	0	1
1	1	1

IL-1 or p53 is needed for ET-1 expression and activation, hence forming an OR relation.

26

IL-1_K	p53_K	SCF_K
0	0	0
0	1	1
1	0	1
1	1	1

IL-1 or p53 is needed for SCF production and activation, hence forming an OR relation.

27

p53_K	a-MSH_K
0	0
1	1

p53 increases the transcriptional activity of a-MSH upon UVB irradiation.

28

IL-1_K	COX2_K	PGE2_K
0	0	0
0	1	0
1	0	0
1	1	1

COX-2 promotes PGE2 production in response to UVB irradiation in an IL-1 dependent manner.

29

ET-1_K	ETR_M
0	0
1	1

ET-1 interacts with and activates ETR.

<table>
<tr><td>30</td><td><table><tr><th>SCF_K</th><th>c-Kit_M</th></tr><tr><td>0</td><td>0</td></tr><tr><td>1</td><td>1</td></tr></table></td><td>SCF binds to and activates c-Kit.</td></tr>
<tr><td>31</td><td><table><tr><th>a-MSH_K</th><th>MC1R_M</th></tr><tr><td>0</td><td>0</td></tr><tr><td>1</td><td>1</td></tr></table></td><td>Binding of a-MSH to the MC1R stimulates activation of MC1R and its downstream signaling proteins.</td></tr>
<tr><td>32</td><td><table><tr><th>PGE2_K</th><th>EP4_M</th></tr><tr><td>0</td><td>0</td></tr><tr><td>1</td><td>1</td></tr></table></td><td>EP4 is a G protein-coupled receptor which activates cAMP signaling in response to PGE2 stimulation.</td></tr>
<tr><td>33</td><td><table><tr><th>ETR_M</th><th>PKC_M</th></tr><tr><td>0</td><td>0</td></tr><tr><td>1</td><td>1</td></tr></table></td><td>ET-1 bound active ETR activates PKC.</td></tr>
<tr><td>34</td><td><table><tr><th>ERK_M</th><th>c-Kit_M</th><th>SG_M</th></tr><tr><td>0</td><td>0</td><td>0</td></tr><tr><td>0</td><td>1</td><td>1</td></tr><tr><td>1</td><td>0</td><td>0</td></tr><tr><td>1</td><td>1</td><td>0</td></tr></table></td><td>c-Kit interacts with SG complex, which in turn activates downstream Ras.
Activated ERK phosphorylates Sos and promotes disassociation of the SG complex.</td></tr>
<tr><td>35</td><td><table><tr><th>MC1R_M</th><th>AC_M</th></tr><tr><td>0</td><td>0</td></tr><tr><td>1</td><td>1</td></tr></table></td><td>a-MSH-bound activated MC1R stimulates AC activation.</td></tr>
<tr><td>36</td><td><table><tr><th>Akt_M</th><th>ASK1_M</th></tr><tr><td>0</td><td>1</td></tr><tr><td>1</td><td>0</td></tr></table></td><td>Akt phosphorylates and negatively regulates ASK1 activity.</td></tr>
<tr><td>37</td><td><table><tr><th>PKC_M</th><th>cAMP_M</th><th>Ras_M</th><th>PI3K_M</th></tr><tr><td>0</td><td>0</td><td>0</td><td>0</td></tr><tr><td>0</td><td>0</td><td>1</td><td>1</td></tr><tr><td>0</td><td>1</td><td>0</td><td>0</td></tr><tr><td>0</td><td>1</td><td>1</td><td>0</td></tr><tr><td>1</td><td>0</td><td>0</td><td>1</td></tr><tr><td>1</td><td>0</td><td>1</td><td>1</td></tr><tr><td>1</td><td>1</td><td>0</td><td>0</td></tr><tr><td>1</td><td>1</td><td>1</td><td>0</td></tr></table></td><td>Ras interacts directly with the catalytic subunit of PI3K in a GTP-dependent manner.
cAMP inhibits PI3K activation, which in turn inactivates downstream Akt kinase.</td></tr>
</table>

38

SG_M	cAMP_M	Ras_M
0	0	0
0	1	1
1	0	1
1	1	1

SG complex catalyzes the exchange of GDP with GTP and induces Ras activation.
SG does not participate in the cAMP-dependent Ras activation, hence forming an OR relation.

39

PDE_M	EP4_M	AC_M	cAMP_M
0	0	0	0
0	0	1	1
0	1	0	1
0	1	1	1
1	0	0	0
1	0	1	0
1	1	0	0
1	1	1	0

The activation of EP4 or AC is required for the conversion of ATP to cAMP.
PDE hydrolyzes and inhibits the cAMP activity, which is dominant to the positive regulators.

40

ASK1_M	Akt_M	MKK4_M
0	0	0
0	1	0
1	0	1
1	1	0

ASK1 activates MKK4.
Akt negatively regulates MKK4 activity by means of phosphorylation.

41

PI3K_M	PDK1_M
0	0
1	1

PI3K promotes the binding of PDK1 with PIP3, which in turn activates downstream kinases.

42

Ras_M	PKC_M	Raf_M
0	0	0
0	1	1
1	0	1
1	1	1

Ras directly interacts with and activates Raf.
Ras binding of Raf promotes conformational changes of Raf that relieve Raf autoinhibition.
PKC phosphorylation rescues the inhibition of Raf by ERK.

43

cAMP_M	PKA_M
0	0
1	1

cAMP activates PKA by binding to its regulatory subunits, causing their dissociation from catalytic subunits.

44	ASK1_M MKK6_M<br>0 0<br>1 1	ASK1 phosphorylates and activates and MKK6.
45	PDK1_M Akt_M<br>0 0<br>1 1	PDK1 phosphorylates Akt to promote its activation.
46	Raf_M MEK_M<br>0 0<br>1 1	Raf phosphorylates MEK, and promotes its activation.
47	PKA_M PDE_M<br>0 0<br>1 1	Phosphorylation of PDE by PKA enhances its activity.
48	MKK6_M p38_M<br>0 0<br>1 1	MKK6 phosphorylates and activates p38.
49	Akt_M GSK3b_M<br>0 1<br>1 0	AKT inhibits kinase activity of GSK3b by phosphorylating GSK3b at serine 9.
50	MEK_M p38_M ERK_M<br>0 0 0<br>0 1 0<br>1 0 1<br>1 1 0	MEK phosphorylates and activates ERK.<br>When both MEK and p38 are activated, dephosphorylation of ERK occurs.
51	p38_M ERK_M MSK_M<br>0 0 0<br>0 1 1<br>1 0 1<br>1 1 1	The MSK activation requires phosphorylation by ERK or p38.
52	MKK4_M JNK_M<br>0 0<br>1 1	Active MKK4 phosphorylates JNK and promotes its activation.

53

Akt_M	MDM2_M
0	0
1	1

Akt promotes nuclear localization and activation of MDM2 by means of phosphorylation.

54

ERK_M	RSK_M
0	0
1	1

RSK is directly phosphorylated by ERK, which promotes its kinase activity.

55

MDM2_M	MITFprotein_M	p38_M	p53_M
0	0	0	0
0	0	1	1
0	1	0	0
0	1	1	0
1	0	0	0
1	0	1	0
1	1	0	0
1	1	1	0

p38 phosphorylates p53 protein at in response to UVB irradiation, leading to p53 activation.
The nuclear localization of p53, which is necessary for its transcriptional activation, is inhibited by direct binding of p53 to MDM2.

56

GSK3b_M	b-catenin_M
0	1
1	0

GSK3b phosphorylates b-catenin and promotes its degradation.

57

Akt_M	PKA_M	MSK_M	CREB_M
0	0	0	0
0	0	1	1
0	1	0	1
0	1	1	1
1	0	0	1
1	0	1	1
1	1	0	1
1	1	1	1

Akt, PKA, or MSK stimulates CREB activity via a serine 133-dependent mechanism.

	p53_M	JNK_M	p38_M	GSK3b_M	b-catenin_M	Akt_M	ERK_M	RSK_M	CREB_M	MITFprotein_M	Bcl-2_M
	0	0	0	0	0	0	0	0	0	0	0
	0	0	0	0	0	0	0	0	0	1	0
	0	0	0	0	0	0	0	0	1	0	0
	0	0	0	0	0	0	0	0	1	1	0
	0	0	0	0	0	0	0	1	0	0	0
	0	0	0	0	0	0	0	1	0	1	1
	0	0	0	0	0	0	0	1	1	0	1
	0	0	0	0	0	0	0	1	1	1	1
	0	0	0	0	0	0	1	0	0	0	0
	0	0	0	0	0	0	1	0	0	1	1
	0	0	0	0	0	0	1	0	1	0	1
	0	0	0	0	0	0	1	0	1	1	1
	0	0	0	0	0	0	1	1	0	0	0
	0	0	0	0	0	0	1	1	0	1	1
	0	0	0	0	0	0	1	1	1	0	1
	0	0	0	0	0	0	1	1	1	1	1
	0	0	0	0	0	1	0	0	0	0	0
	0	0	0	0	0	1	0	0	0	1	1
	0	0	0	0	0	1	0	0	1	0	1
	0	0	0	0	0	1	0	0	1	1	1
	0	0	0	0	0	1	0	1	0	0	0
	0	0	0	0	0	1	0	1	0	1	1
	0	0	0	0	0	1	0	1	1	0	1
	0	0	0	0	0	1	0	1	1	1	1
	0	0	0	0	0	1	1	0	0	0	0
	0	0	0	0	0	1	1	0	0	1	1
	0	0	0	0	0	1	1	0	1	0	1
	0	0	0	0	0	1	1	0	1	1	1
	0	0	0	0	0	1	1	1	0	0	1
	0	0	0	0	0	1	1	1	0	1	1
	0	0	0	0	0	1	1	1	1	0	1
58	0	0	0	0	0	1	1	1	1	1	1
	0	0	0	0	1	0	0	0	0	0	0
	0	0	0	0	1	0	0	0	0	1	0
	0	0	0	0	1	0	0	0	1	0	0
	0	0	0	0	1	0	0	0	1	1	0
	0	0	0	0	1	0	0	1	0	0	0
	0	0	0	0	1	0	0	1	0	1	1
	0	0	0	0	1	0	0	1	1	0	1
	0	0	0	0	1	0	0	1	1	1	1
	0	0	0	0	1	0	1	0	0	0	0
	0	0	0	0	1	0	1	0	0	1	1
	0	0	0	0	1	0	1	0	1	0	1
	0	0	0	0	1	0	1	0	1	1	1
	0	0	0	0	1	0	1	1	0	0	0
	0	0	0	0	1	0	1	1	0	1	1
	0	0	0	0	1	0	1	1	1	0	1
	0	0	0	0	1	0	1	1	1	1	1
	0	0	0	0	1	1	0	0	0	0	0
	0	0	0	0	1	1	0	0	0	1	1
	0	0	0	0	1	1	0	0	1	0	1
	0	0	0	0	1	1	0	0	1	1	1
	0	0	0	0	1	1	0	1	0	0	0
	0	0	0	0	1	1	0	1	0	1	1
	0	0	0	0	1	1	0	1	1	0	1
	0	0	0	0	1	1	0	1	1	1	1
	0	0	0	0	1	1	1	0	0	0	0
	0	0	0	0	1	1	1	0	0	1	1
	0	0	0	0	1	1	1	0	1	0	1
	0	0	0	0	1	1	1	0	1	1	1
	0	0	0	0	1	1	1	1	0	0	1
	0	0	0	0	1	1	1	1	0	1	1
	0	0	0	0	1	1	1	1	1	0	1
	0	0	0	0	1	1	1	1	1	1	1

CREB or MITF is required for the transcriptional activation of Bcl-2 gene.

Phosphorylation of BAD (a Bcl-2 antagonist) by Akt, ERK, or RSK is needed for the full activation of Bcl-2 protein.

Beta-catenin and GSK3-beta upregulates Bcl-2 expression level.

Bcl-2 activity is suppressed if any of its negative regulators is activated.

Hence, the activation condition for Bcl-2_M is 'AND(OR(MITFprotein_M, CREB_M), OR(Akt_M, ERK_M, RSK_M, b-catenin_M, GSK3b_M), NOT(OR(p53_M, JNK_M, p38_M)))'.

	0	0	0	1	0	0	0	0	0	0	0
	0	0	0	1	0	0	0	0	0	1	0
	0	0	0	1	0	0	0	0	1	0	0
	0	0	0	1	0	0	0	0	1	1	0
	0	0	0	1	0	0	0	1	0	0	0
	0	0	0	1	0	0	0	1	0	1	1
	0	0	0	1	0	0	0	1	1	0	1
	0	0	0	1	0	0	0	1	1	1	1
	0	0	0	1	0	0	1	0	0	0	0
	0	0	0	1	0	0	1	0	0	1	1
	0	0	0	1	0	0	1	0	1	0	1
	0	0	0	1	0	0	1	0	1	1	1
	0	0	0	1	0	0	1	1	0	0	0
	0	0	0	1	0	0	1	1	0	1	1
	0	0	0	1	0	0	1	1	1	0	1
	0	0	0	1	0	0	1	1	1	1	1
	0	0	0	1	0	1	0	0	0	0	0
	0	0	0	1	0	1	0	0	0	1	1
	0	0	0	1	0	1	0	0	1	0	1
	0	0	0	1	0	1	0	0	1	1	1
	0	0	0	1	0	1	0	1	0	0	0
	0	0	0	1	0	1	0	1	0	1	1
	0	0	0	1	0	1	0	1	1	0	1
	0	0	0	1	0	1	0	1	1	1	1
	0	0	0	1	0	1	1	0	0	0	0
	0	0	0	1	0	1	1	0	0	1	1
	0	0	0	1	0	1	1	0	1	0	1
	0	0	0	1	0	1	1	0	1	1	1
	0	0	0	1	0	1	1	1	0	0	1
	0	0	0	1	0	1	1	1	0	1	1
	0	0	0	1	0	1	1	1	1	0	1
	0	0	0	1	0	1	1	1	1	1	1
58	0	0	0	1	1	0	0	0	0	0	0
	0	0	0	1	1	0	0	0	0	1	0
	0	0	0	1	1	0	0	0	1	0	0
	0	0	0	1	1	0	0	0	1	1	0
	0	0	0	1	1	0	0	1	0	0	0
	0	0	0	1	1	0	0	1	0	1	1
	0	0	0	1	1	0	0	1	1	0	1
	0	0	0	1	1	0	0	1	1	1	1
	0	0	0	1	1	0	1	0	0	0	0
	0	0	0	1	1	0	1	0	0	1	1
	0	0	0	1	1	0	1	0	1	0	1
	0	0	0	1	1	0	1	0	1	1	1
	0	0	0	1	1	0	1	1	0	0	0
	0	0	0	1	1	0	1	1	0	1	1
	0	0	0	1	1	0	1	1	1	0	1
	0	0	0	1	1	0	1	1	1	1	1
	0	0	0	1	1	1	0	0	0	0	0
	0	0	0	1	1	1	0	0	0	1	1
	0	0	0	1	1	1	0	0	1	0	1
	0	0	0	1	1	1	0	0	1	1	1
	0	0	0	1	1	1	0	1	0	0	0
	0	0	0	1	1	1	0	1	0	1	1
	0	0	0	1	1	1	0	1	1	0	1
	0	0	0	1	1	1	0	1	1	1	1
	0	0	0	1	1	1	1	0	0	0	0
	0	0	0	1	1	1	1	0	0	1	1
	0	0	0	1	1	1	1	0	1	0	1
	0	0	0	1	1	1	1	0	1	1	1
	0	0	0	1	1	1	1	1	0	0	1
	0	0	0	1	1	1	1	1	0	1	1
	0	0	0	1	1	1	1	1	1	0	1
	0	0	0	1	1	1	1	1	1	1	1
	0	0	1	0	0	0	0	0	0	0	0

CREB or MITF is required for the transcriptional activation of Bcl-2 gene.
Phosphorylation of BAD (a Bcl-2 antagonist) by Akt, ERK, or RSK is needed for the full activation of Bcl-2 protein.
Beta-catenin and GSK3-beta upregulates Bcl-2 expression level.
Bcl-2 activity is suppressed if any of its negative regulators is activated.
Hence, the activation condition for Bcl-2_M is 'AND(OR(MITFprotein_M, CREB_M), OR(Akt_M, ERK_M, RSK_M, b-catenin_M, GSK3b_M), NOT(OR(p53_M, JNK_M, p38_M)))'.

	0	0	1	0	0	0	0	0	0	1	0	
	0	0	1	0	0	0	0	0	1	0	0	
	0	0	1	0	0	0	0	0	1	1	0	
	0	0	1	0	0	0	0	1	0	0	0	
	0	0	1	0	0	0	0	1	0	1	0	
	0	0	1	0	0	0	0	1	1	0	0	
	0	0	1	0	0	0	0	1	1	1	0	
	0	0	1	0	0	0	1	0	0	0	0	
	0	0	1	0	0	0	1	0	0	1	0	
	0	0	1	0	0	0	1	0	1	0	0	
	0	0	1	0	0	0	1	0	1	1	0	
	0	0	1	0	0	0	1	1	0	0	0	
	0	0	1	0	0	0	1	1	0	1	0	
	0	0	1	0	0	0	1	1	1	0	0	
	0	0	1	0	0	0	1	1	1	1	0	
	0	0	1	0	0	1	0	0	0	0	0	
	0	0	1	0	0	1	0	0	0	1	0	
	0	0	1	0	0	1	0	0	1	0	0	
	0	0	1	0	0	1	0	0	1	1	0	
	0	0	1	0	0	1	0	1	0	0	0	
	0	0	1	0	0	1	0	1	0	1	0	
	0	0	1	0	0	1	0	1	1	0	0	
	0	0	1	0	0	1	0	1	1	1	0	
	0	0	1	0	0	1	1	0	0	0	0	
	0	0	1	0	0	1	1	0	0	1	0	
	0	0	1	0	0	1	1	0	1	0	0	
	0	0	1	0	0	1	1	0	1	1	0	
	0	0	1	0	0	1	1	1	0	0	0	
	0	0	1	0	0	1	1	1	0	1	0	
	0	0	1	0	0	1	1	1	1	0	0	
	0	0	1	0	0	1	1	1	1	1	0	
	0	0	1	0	1	0	0	0	0	0	0	
58	0	0	1	0	1	0	0	0	0	1	0	CREB or MITF is required for the transcriptional activation of Bcl-2 gene.<br>Phosphorylation of BAD (a Bcl-2 antagonist) by Akt, ERK, or RSK is needed for the full activation of Bcl-2 protein.<br>Beta-catenin and GSK3-beta upregulates Bcl-2 expression level.<br>Bcl-2 activity is suppressed if any of its negative regulators is activated.<br>Hence, the activation condition for Bcl-2_M is 'AND(OR(MITFprotein_M, CREB_M), OR(Akt_M, ERK_M, RSK_M, b-catenin_M, GSK3b_M), NOT(OR(p53_M, JNK_M, p38_M)))'.
	0	0	1	0	1	0	0	0	1	0	0	
	0	0	1	0	1	0	0	0	1	1	0	
	0	0	1	0	1	0	0	1	0	0	0	
	0	0	1	0	1	0	0	1	0	1	0	
	0	0	1	0	1	0	0	1	1	0	0	
	0	0	1	0	1	0	0	1	1	1	0	
	0	0	1	0	1	0	1	0	0	0	0	
	0	0	1	0	1	0	1	0	0	1	0	
	0	0	1	0	1	0	1	0	1	0	0	
	0	0	1	0	1	0	1	0	1	1	0	
	0	0	1	0	1	0	1	1	0	0	0	
	0	0	1	0	1	0	1	1	0	1	0	
	0	0	1	0	1	0	1	1	1	0	0	
	0	0	1	0	1	0	1	1	1	1	0	
	0	0	1	0	1	1	0	0	0	0	0	
	0	0	1	0	1	1	0	0	0	1	0	
	0	0	1	0	1	1	0	0	1	0	0	
	0	0	1	0	1	1	0	0	1	1	0	
	0	0	1	0	1	1	0	1	0	0	0	
	0	0	1	0	1	1	0	1	0	1	0	
	0	0	1	0	1	1	0	1	1	0	0	
	0	0	1	0	1	1	0	1	1	1	0	
	0	0	1	0	1	1	1	0	0	0	0	
	0	0	1	0	1	1	1	0	0	1	0	
	0	0	1	0	1	1	1	0	1	0	0	
	0	0	1	0	1	1	1	0	1	1	0	
	0	0	1	0	1	1	1	1	0	0	0	
	0	0	1	0	1	1	1	1	0	1	0	
	0	0	1	0	1	1	1	1	1	0	0	
	0	0	1	0	1	1	1	1	1	1	0	
	0	0	1	1	0	0	0	0	0	0	0	
	0	0	1	1	0	0	0	0	0	1	0	

	0	0	1	1	0	0	0	0	1	0	0	CREB or MITF is required for the transcriptional activation of Bcl-2 gene.<br>Phosphorylation of BAD (a Bcl-2 antagonist) by Akt, ERK, or RSK is needed for the full activation of Bcl-2 protein.<br>Beta-catenin and GSK3-beta upregulates Bcl-2 expression level.<br>Bcl-2 activity is suppressed if any of its negative regulators is activated.<br>Hence, the activation condition for Bcl-2_M is 'AND(OR(MITFprotein_M, CREB_M), OR(Akt_M, ERK_M, RSK_M, b-catenin_M, GSK3b_M), NOT(OR(p53_M, JNK_M, p38_M)))'.
	0	0	1	1	0	0	0	0	1	1	0	
	0	0	1	1	0	0	0	1	0	0	0	
	0	0	1	1	0	0	0	1	0	1	0	
	0	0	1	1	0	0	0	1	1	0	0	
	0	0	1	1	0	0	0	1	1	1	0	
	0	0	1	1	0	0	1	0	0	0	0	
	0	0	1	1	0	0	1	0	0	1	0	
	0	0	1	1	0	0	1	0	1	0	0	
	0	0	1	1	0	0	1	0	1	1	0	
	0	0	1	1	0	0	1	1	0	0	0	
	0	0	1	1	0	0	1	1	0	1	0	
	0	0	1	1	0	0	1	1	1	0	0	
	0	0	1	1	0	0	1	1	1	1	0	
	0	0	1	1	0	1	0	0	0	0	0	
	0	0	1	1	0	1	0	0	0	1	0	
	0	0	1	1	0	1	0	0	1	0	0	
	0	0	1	1	0	1	0	0	1	1	0	
	0	0	1	1	0	1	0	1	0	0	0	
	0	0	1	1	0	1	0	1	0	1	0	
	0	0	1	1	0	1	0	1	1	0	0	
	0	0	1	1	0	1	0	1	1	1	0	
	0	0	1	1	0	1	1	0	0	0	0	
	0	0	1	1	0	1	1	0	0	1	0	
	0	0	1	1	0	1	1	0	1	0	0	
	0	0	1	1	0	1	1	0	1	1	0	
	0	0	1	1	0	1	1	1	0	0	0	
	0	0	1	1	0	1	1	1	0	1	0	
	0	0	1	1	0	1	1	1	1	0	0	
	0	0	1	1	0	1	1	1	1	1	0	
	0	0	1	1	1	0	0	0	0	0	0	
	0	0	1	1	1	0	0	0	0	1	0	
58	0	0	1	1	1	0	0	0	1	0	0	
	0	0	1	1	1	0	0	0	1	1	0	
	0	0	1	1	1	0	0	1	0	0	0	
	0	0	1	1	1	0	0	1	0	1	0	
	0	0	1	1	1	0	0	1	1	0	0	
	0	0	1	1	1	0	0	1	1	1	0	
	0	0	1	1	1	0	1	0	0	0	0	
	0	0	1	1	1	0	1	0	0	1	0	
	0	0	1	1	1	0	1	0	1	0	0	
	0	0	1	1	1	0	1	0	1	1	0	
	0	0	1	1	1	0	1	1	0	0	0	
	0	0	1	1	1	0	1	1	0	1	0	
	0	0	1	1	1	0	1	1	1	0	0	
	0	0	1	1	1	0	1	1	1	1	0	
	0	0	1	1	1	1	0	0	0	0	0	
	0	0	1	1	1	1	0	0	0	1	0	
	0	0	1	1	1	1	0	0	1	0	0	
	0	0	1	1	1	1	0	0	1	1	0	
	0	0	1	1	1	1	0	1	0	0	0	
	0	0	1	1	1	1	0	1	0	1	0	
	0	0	1	1	1	1	0	1	1	0	0	
	0	0	1	1	1	1	0	1	1	1	0	
	0	0	1	1	1	1	1	0	0	0	0	
	0	0	1	1	1	1	1	0	0	1	0	
	0	0	1	1	1	1	1	0	1	0	0	
	0	0	1	1	1	1	1	0	1	1	0	
	0	0	1	1	1	1	1	1	0	0	0	
	0	0	1	1	1	1	1	1	0	1	0	
	0	0	1	1	1	1	1	1	1	0	0	
	0	0	1	1	1	1	1	1	1	1	0	
	0	1	0	0	0	0	0	0	0	0	0	
	0	1	0	0	0	0	0	0	0	1	0	
	0	1	0	0	0	0	0	0	1	0	0	

	0	1	0	0	0	0	0	0	1	1	0	
	0	1	0	0	0	0	0	1	0	0	0	
	0	1	0	0	0	0	0	1	0	1	0	
	0	1	0	0	0	0	0	1	1	0	0	
	0	1	0	0	0	0	0	1	1	1	0	
	0	1	0	0	0	0	1	0	0	0	0	
	0	1	0	0	0	0	1	0	0	1	0	
	0	1	0	0	0	0	1	0	1	0	0	
	0	1	0	0	0	0	1	0	1	1	0	
	0	1	0	0	0	0	1	1	0	0	0	
	0	1	0	0	0	0	1	1	0	1	0	
	0	1	0	0	0	0	1	1	1	0	0	
	0	1	0	0	0	0	1	1	1	1	0	
	0	1	0	0	0	1	0	0	0	0	0	
	0	1	0	0	0	1	0	0	0	1	0	
	0	1	0	0	0	1	0	0	1	0	0	CREB or MITF is required for the transcriptional activation of Bcl-2 gene.<br>Phosphorylation of BAD (a Bcl-2 antagonist) by Akt, ERK, or RSK is needed for the full activation of Bcl-2 protein.<br>Beta-catenin and GSK3-beta upregulates Bcl-2 expression level.<br>Bcl-2 activity is suppressed if any of its negative regulators is activated.<br>Hence, the activation condition for Bcl-2_M is 'AND(OR(MITFprotein_M, CREB_M), OR(Akt_M, ERK_M, RSK_M, b-catenin_M, GSK3b_M), NOT(OR(p53_M, JNK_M, p38_M)))'.
	0	1	0	0	0	1	0	0	1	1	0	
	0	1	0	0	0	1	0	1	0	0	0	
	0	1	0	0	0	1	0	1	0	1	0	
	0	1	0	0	0	1	0	1	1	0	0	
	0	1	0	0	0	1	0	1	1	1	0	
	0	1	0	0	0	1	1	0	0	0	0	
	0	1	0	0	0	1	1	0	0	1	0	
	0	1	0	0	0	1	1	0	1	0	0	
	0	1	0	0	0	1	1	0	1	1	0	
	0	1	0	0	0	1	1	1	0	0	0	
	0	1	0	0	0	1	1	1	0	1	0	
	0	1	0	0	0	1	1	1	1	0	0	
	0	1	0	0	0	1	1	1	1	1	0	
	0	1	0	0	1	0	0	0	0	0	0	
	0	1	0	0	1	0	0	0	0	1	0	
	0	1	0	0	1	0	0	0	1	0	0	
58	0	1	0	0	1	0	0	0	1	1	0	
	0	1	0	0	1	0	0	1	0	0	0	
	0	1	0	0	1	0	0	1	0	1	0	
	0	1	0	0	1	0	0	1	1	0	0	
	0	1	0	0	1	0	0	1	1	1	0	
	0	1	0	0	1	0	1	0	0	0	0	
	0	1	0	0	1	0	1	0	0	1	0	
	0	1	0	0	1	0	1	0	1	0	0	
	0	1	0	0	1	0	1	0	1	1	0	
	0	1	0	0	1	0	1	1	0	0	0	
	0	1	0	0	1	0	1	1	0	1	0	
	0	1	0	0	1	0	1	1	1	0	0	
	0	1	0	0	1	0	1	1	1	1	0	
	0	1	0	0	1	1	0	0	0	0	0	
	0	1	0	0	1	1	0	0	0	1	0	
	0	1	0	0	1	1	0	0	1	0	0	
	0	1	0	0	1	1	0	0	1	1	0	
	0	1	0	0	1	1	0	1	0	0	0	
	0	1	0	0	1	1	0	1	0	1	0	
	0	1	0	0	1	1	0	1	1	0	0	
	0	1	0	0	1	1	0	1	1	1	0	
	0	1	0	0	1	1	1	0	0	0	0	
	0	1	0	0	1	1	1	0	0	1	0	
	0	1	0	0	1	1	1	0	1	0	0	
	0	1	0	0	1	1	1	0	1	1	0	
	0	1	0	0	1	1	1	1	0	0	0	
	0	1	0	0	1	1	1	1	0	1	0	
	0	1	0	0	1	1	1	1	1	0	0	
	0	1	0	0	1	1	1	1	1	1	0	
	0	1	0	1	0	0	0	0	0	0	0	
	0	1	0	1	0	0	0	0	0	1	0	
	0	1	0	1	0	0	0	0	1	0	0	
	0	1	0	1	0	0	0	0	1	1	0	

	0	1	0	1	0	0	0	1	0	0	0	
	0	1	0	1	0	0	0	1	0	1	0	
	0	1	0	1	0	0	0	1	1	0	0	
	0	1	0	1	0	0	0	1	1	1	0	
	0	1	0	1	0	0	1	0	0	0	0	
	0	1	0	1	0	0	1	0	0	1	0	
	0	1	0	1	0	0	1	0	1	0	0	
	0	1	0	1	0	0	1	0	1	1	0	
	0	1	0	1	0	0	1	1	0	0	0	
	0	1	0	1	0	0	1	1	0	1	0	
	0	1	0	1	0	0	1	1	1	0	0	
	0	1	0	1	0	0	1	1	1	1	0	
	0	1	0	1	0	1	0	0	0	0	0	
	0	1	0	1	0	1	0	0	0	1	0	
	0	1	0	1	0	1	0	0	1	0	0	
	0	1	0	1	0	1	0	0	1	1	0	CREB or MITF is required for the transcriptional activation of Bcl-2 gene.<br>Phosphorylation of BAD (a Bcl-2 antagonist) by Akt, ERK, or RSK is needed for the full activation of Bcl-2 protein.<br>Beta-catenin and GSK3-beta upregulates Bcl-2 expression level.<br>Bcl-2 activity is suppressed if any of its negative regulators is activated.<br>Hence, the activation condition for Bcl-2_M is 'AND(OR(MITFprotein_M, CREB_M), OR(Akt_M, ERK_M, RSK_M, b-catenin_M, GSK3b_M), NOT(OR(p53_M, JNK_M, p38_M)))'.
	0	1	0	1	0	1	0	1	0	0	0	
	0	1	0	1	0	1	0	1	0	1	0	
	0	1	0	1	0	1	0	1	1	0	0	
	0	1	0	1	0	1	0	1	1	1	0	
	0	1	0	1	0	1	1	0	0	0	0	
	0	1	0	1	0	1	1	0	0	1	0	
	0	1	0	1	0	1	1	0	1	0	0	
	0	1	0	1	0	1	1	0	1	1	0	
	0	1	0	1	0	1	1	1	0	0	0	
	0	1	0	1	0	1	1	1	0	1	0	
	0	1	0	1	0	1	1	1	1	0	0	
	0	1	0	1	0	1	1	1	1	1	0	
	0	1	0	1	1	0	0	0	0	0	0	
	0	1	0	1	1	0	0	0	0	1	0	
	0	1	0	1	1	0	0	0	1	0	0	
	0	1	0	1	1	0	0	0	1	1	0	
58	0	1	0	1	1	0	0	1	0	0	0	
	0	1	0	1	1	0	0	1	0	1	0	
	0	1	0	1	1	0	0	1	1	0	0	
	0	1	0	1	1	0	0	1	1	1	0	
	0	1	0	1	1	0	1	0	0	0	0	
	0	1	0	1	1	0	1	0	0	1	0	
	0	1	0	1	1	0	1	0	1	0	0	
	0	1	0	1	1	0	1	0	1	1	0	
	0	1	0	1	1	0	1	1	0	0	0	
	0	1	0	1	1	0	1	1	0	1	0	
	0	1	0	1	1	0	1	1	1	0	0	
	0	1	0	1	1	0	1	1	1	1	0	
	0	1	0	1	1	1	0	0	0	0	0	
	0	1	0	1	1	1	0	0	0	1	0	
	0	1	0	1	1	1	0	0	1	0	0	
	0	1	0	1	1	1	0	0	1	1	0	
	0	1	0	1	1	1	0	1	0	0	0	
	0	1	0	1	1	1	0	1	0	1	0	
	0	1	0	1	1	1	0	1	1	0	0	
	0	1	0	1	1	1	0	1	1	1	0	
	0	1	0	1	1	1	1	0	0	0	0	
	0	1	0	1	1	1	1	0	0	1	0	
	0	1	0	1	1	1	1	0	1	0	0	
	0	1	0	1	1	1	1	0	1	1	0	
	0	1	0	1	1	1	1	1	0	0	0	
	0	1	0	1	1	1	1	1	0	1	0	
	0	1	0	1	1	1	1	1	1	0	0	
	0	1	0	1	1	1	1	1	1	1	0	
	0	1	1	0	0	0	0	0	0	0	0	
	0	1	1	0	0	0	0	0	0	1	0	
	0	1	1	0	0	0	0	0	1	0	0	
	0	1	1	0	0	0	0	0	1	1	0	
	0	1	1	0	0	0	0	1	0	0	0	

	0	1	1	0	0	0	0	1	0	1	0	
	0	1	1	0	0	0	0	1	1	0	0	
	0	1	1	0	0	0	0	1	1	1	0	
	0	1	1	0	0	0	1	0	0	0	0	
	0	1	1	0	0	0	1	0	0	1	0	
	0	1	1	0	0	0	1	0	1	0	0	
	0	1	1	0	0	0	1	0	1	1	0	
	0	1	1	0	0	0	1	1	0	0	0	
	0	1	1	0	0	0	1	1	0	1	0	
	0	1	1	0	0	0	1	1	1	0	0	
	0	1	1	0	0	0	1	1	1	1	0	
	0	1	1	0	0	1	0	0	0	0	0	
	0	1	1	0	0	1	0	0	0	1	0	
	0	1	1	0	0	1	0	0	1	0	0	
	0	1	1	0	0	1	0	0	1	1	0	
	0	1	1	0	0	1	0	1	0	0	0	
	0	1	1	0	0	1	0	1	0	1	0	
	0	1	1	0	0	1	0	1	1	0	0	
	0	1	1	0	0	1	0	1	1	1	0	
	0	1	1	0	0	1	1	0	0	0	0	
	0	1	1	0	0	1	1	0	0	1	0	
	0	1	1	0	0	1	1	0	1	0	0	
	0	1	1	0	0	1	1	0	1	1	0	
	0	1	1	0	0	1	1	1	0	0	0	
	0	1	1	0	0	1	1	1	0	1	0	
	0	1	1	0	0	1	1	1	1	0	0	
	0	1	1	0	0	1	1	1	1	1	0	
	0	1	1	0	1	0	0	0	0	0	0	
	0	1	1	0	1	0	0	0	0	1	0	
	0	1	1	0	1	0	0	0	1	0	0	
	0	1	1	0	1	0	0	0	1	1	0	
	0	1	1	0	1	0	0	1	0	0	0	
58	0	1	1	0	1	0	0	1	0	1	0	CREB or MITF is required for the transcriptional activation of Bcl-2 gene.<br>Phosphorylation of BAD (a Bcl-2 antagonist) by Akt, ERK, or RSK is needed for the full activation of Bcl-2 protein.<br>Beta-catenin and GSK3-beta upregulates Bcl-2 expression level.<br>Bcl-2 activity is suppressed if any of its negative regulators is activated.<br>Hence, the activation condition for Bcl-2_M is 'AND(OR(MITFprotein_M, CREB_M), OR(Akt_M, ERK_M, RSK_M, b-catenin_M, GSK3b_M), NOT(OR(p53_M, JNK_M, p38_M)))'.
	0	1	1	0	1	0	0	1	1	0	0	
	0	1	1	0	1	0	0	1	1	1	0	
	0	1	1	0	1	0	1	0	0	0	0	
	0	1	1	0	1	0	1	0	0	1	0	
	0	1	1	0	1	0	1	0	1	0	0	
	0	1	1	0	1	0	1	0	1	1	0	
	0	1	1	0	1	0	1	1	0	0	0	
	0	1	1	0	1	0	1	1	0	1	0	
	0	1	1	0	1	0	1	1	1	0	0	
	0	1	1	0	1	0	1	1	1	1	0	
	0	1	1	0	1	1	0	0	0	0	0	
	0	1	1	0	1	1	0	0	0	1	0	
	0	1	1	0	1	1	0	0	1	0	0	
	0	1	1	0	1	1	0	0	1	1	0	
	0	1	1	0	1	1	0	1	0	0	0	
	0	1	1	0	1	1	0	1	0	1	0	
	0	1	1	0	1	1	0	1	1	0	0	
	0	1	1	0	1	1	0	1	1	1	0	
	0	1	1	0	1	1	1	0	0	0	0	
	0	1	1	0	1	1	1	0	0	1	0	
	0	1	1	0	1	1	1	0	1	0	0	
	0	1	1	0	1	1	1	0	1	1	0	
	0	1	1	0	1	1	1	1	0	0	0	
	0	1	1	0	1	1	1	1	0	1	0	
	0	1	1	0	1	1	1	1	1	0	0	
	0	1	1	0	1	1	1	1	1	1	0	
	0	1	1	1	0	0	0	0	0	0	0	
	0	1	1	1	0	0	0	0	0	1	0	
	0	1	1	1	0	0	0	0	1	0	0	
	0	1	1	1	0	0	0	0	1	1	0	
	0	1	1	1	0	0	0	1	0	0	0	
	0	1	1	1	0	0	0	1	0	1	0	

	0	1	1	1	0	0	0	1	1	0	0	
	0	1	1	1	0	0	0	1	1	1	0	
	0	1	1	1	0	0	1	0	0	0	0	
	0	1	1	1	0	0	1	0	0	1	0	
	0	1	1	1	0	0	1	0	1	0	0	
	0	1	1	1	0	0	1	0	1	1	0	
	0	1	1	1	0	0	1	1	0	0	0	
	0	1	1	1	0	0	1	1	0	1	0	
	0	1	1	1	0	0	1	1	1	0	0	
	0	1	1	1	0	0	1	1	1	1	0	
	0	1	1	1	0	1	0	0	0	0	0	
	0	1	1	1	0	1	0	0	0	1	0	
	0	1	1	1	0	1	0	0	1	0	0	
	0	1	1	1	0	1	0	0	1	1	0	
	0	1	1	1	0	1	0	1	0	0	0	
	0	1	1	1	0	1	0	1	0	1	0	
	0	1	1	1	0	1	0	1	1	0	0	
	0	1	1	1	0	1	0	1	1	1	0	
	0	1	1	1	0	1	1	0	0	0	0	
	0	1	1	1	0	1	1	0	0	1	0	
	0	1	1	1	0	1	1	0	1	0	0	
	0	1	1	1	0	1	1	0	1	1	0	
	0	1	1	1	0	1	1	1	0	0	0	
	0	1	1	1	0	1	1	1	0	1	0	
	0	1	1	1	0	1	1	1	1	0	0	
	0	1	1	1	0	1	1	1	1	1	0	
	0	1	1	1	1	0	0	0	0	0	0	
	0	1	1	1	1	0	0	0	0	1	0	
	0	1	1	1	1	0	0	0	1	0	0	
	0	1	1	1	1	0	0	0	1	1	0	
	0	1	1	1	1	0	0	1	0	0	0	
	0	1	1	1	1	0	0	1	0	1	0	
58	0	1	1	1	1	0	0	1	1	0	0	CREB or MITF is required for the transcriptional activation of Bcl-2 gene.<br>Phosphorylation of BAD (a Bcl-2 antagonist) by Akt, ERK, or RSK is needed for the full activation of Bcl-2 protein.<br>Beta-catenin and GSK3-beta upregulates Bcl-2 expression level.<br>Bcl-2 activity is suppressed if any of its negative regulators is activated.<br>Hence, the activation condition for Bcl-2_M is 'AND(OR(MITFprotein_M, CREB_M), OR(Akt_M, ERK_M, RSK_M, b-catenin_M, GSK3b_M), NOT(OR(p53_M, JNK_M, p38_M)))'.
	0	1	1	1	1	0	0	1	1	1	0	
	0	1	1	1	1	0	1	0	0	0	0	
	0	1	1	1	1	0	1	0	0	1	0	
	0	1	1	1	1	0	1	0	1	0	0	
	0	1	1	1	1	0	1	0	1	1	0	
	0	1	1	1	1	0	1	1	0	0	0	
	0	1	1	1	1	0	1	1	0	1	0	
	0	1	1	1	1	0	1	1	1	0	0	
	0	1	1	1	1	0	1	1	1	1	0	
	0	1	1	1	1	1	0	0	0	0	0	
	0	1	1	1	1	1	0	0	0	1	0	
	0	1	1	1	1	1	0	0	1	0	0	
	0	1	1	1	1	1	0	0	1	1	0	
	0	1	1	1	1	1	0	1	0	0	0	
	0	1	1	1	1	1	0	1	0	1	0	
	0	1	1	1	1	1	0	1	1	0	0	
	0	1	1	1	1	1	0	1	1	1	0	
	0	1	1	1	1	1	1	0	0	0	0	
	0	1	1	1	1	1	1	0	0	1	0	
	0	1	1	1	1	1	1	0	1	0	0	
	0	1	1	1	1	1	1	0	1	1	0	
	0	1	1	1	1	1	1	1	0	0	0	
	0	1	1	1	1	1	1	1	0	1	0	
	0	1	1	1	1	1	1	1	1	0	0	
	0	1	1	1	1	1	1	1	1	1	0	
	1	0	0	0	0	0	0	0	0	0	0	
	1	0	0	0	0	0	0	0	0	1	0	
	1	0	0	0	0	0	0	0	1	0	0	
	1	0	0	0	0	0	0	0	1	1	0	
	1	0	0	0	0	0	0	1	0	0	0	
	1	0	0	0	0	0	0	1	0	1	0	
	1	0	0	0	0	0	0	1	1	0	0	

	1	0	0	0	0	0	0	1	1	1	0	
	1	0	0	0	0	0	1	0	0	0	0	
	1	0	0	0	0	0	1	0	0	1	0	
	1	0	0	0	0	0	1	0	1	0	0	
	1	0	0	0	0	0	1	0	1	1	0	
	1	0	0	0	0	0	1	1	0	0	0	
	1	0	0	0	0	0	1	1	0	1	0	
	1	0	0	0	0	0	1	1	1	0	0	
	1	0	0	0	0	0	1	1	1	1	0	
	1	0	0	0	0	1	0	0	0	0	0	
	1	0	0	0	0	1	0	0	0	1	0	
	1	0	0	0	0	1	0	0	1	0	0	
	1	0	0	0	0	1	0	0	1	1	0	
	1	0	0	0	0	1	0	1	0	0	0	
	1	0	0	0	0	1	0	1	0	1	0	
	1	0	0	0	0	1	0	1	1	0	0	CREB or MITF is required for the transcriptional activation of Bcl-2 gene.<br>Phosphorylation of BAD (a Bcl-2 antagonist) by Akt, ERK, or RSK is needed for the full activation of Bcl-2 protein.<br>Beta-catenin and GSK3-beta upregulates Bcl-2 expression level.<br>Bcl-2 activity is suppressed if any of its negative regulators is activated.<br>Hence, the activation condition for Bcl-2_M is 'AND(OR(MITFprotein_M, CREB_M), OR(Akt_M, ERK_M, RSK_M, b-catenin_M, GSK3b_M), NOT(OR(p53_M, JNK_M, p38_M)))'.
	1	0	0	0	0	1	0	1	1	1	0	
	1	0	0	0	0	1	1	0	0	0	0	
	1	0	0	0	0	1	1	0	0	1	0	
	1	0	0	0	0	1	1	0	1	0	0	
	1	0	0	0	0	1	1	0	1	1	0	
	1	0	0	0	0	1	1	1	0	0	0	
	1	0	0	0	0	1	1	1	0	1	0	
	1	0	0	0	0	1	1	1	1	0	0	
	1	0	0	0	0	1	1	1	1	1	0	
	1	0	0	0	1	0	0	0	0	0	0	
	1	0	0	0	1	0	0	0	0	1	0	
	1	0	0	0	1	0	0	0	1	0	0	
	1	0	0	0	1	0	0	0	1	1	0	
	1	0	0	0	1	0	0	1	0	0	0	
	1	0	0	0	1	0	0	1	0	1	0	
	1	0	0	0	1	0	0	1	1	0	0	
58	1	0	0	0	1	0	0	1	1	1	0	
	1	0	0	0	1	0	1	0	0	0	0	
	1	0	0	0	1	0	1	0	0	1	0	
	1	0	0	0	1	0	1	0	1	0	0	
	1	0	0	0	1	0	1	0	1	1	0	
	1	0	0	0	1	0	1	1	0	0	0	
	1	0	0	0	1	0	1	1	0	1	0	
	1	0	0	0	1	0	1	1	1	0	0	
	1	0	0	0	1	0	1	1	1	1	0	
	1	0	0	0	1	1	0	0	0	0	0	
	1	0	0	0	1	1	0	0	0	1	0	
	1	0	0	0	1	1	0	0	1	0	0	
	1	0	0	0	1	1	0	0	1	1	0	
	1	0	0	0	1	1	0	1	0	0	0	
	1	0	0	0	1	1	0	1	0	1	0	
	1	0	0	0	1	1	0	1	1	0	0	
	1	0	0	0	1	1	0	1	1	1	0	
	1	0	0	0	1	1	1	0	0	0	0	
	1	0	0	0	1	1	1	0	0	1	0	
	1	0	0	0	1	1	1	0	1	0	0	
	1	0	0	0	1	1	1	0	1	1	0	
	1	0	0	0	1	1	1	1	0	0	0	
	1	0	0	0	1	1	1	1	0	1	0	
	1	0	0	0	1	1	1	1	1	0	0	
	1	0	0	0	1	1	1	1	1	1	0	
	1	0	0	1	0	0	0	0	0	0	0	
	1	0	0	1	0	0	0	0	0	1	0	
	1	0	0	1	0	0	0	0	1	0	0	
	1	0	0	1	0	0	0	0	1	1	0	
	1	0	0	1	0	0	0	1	0	0	0	
	1	0	0	1	0	0	0	1	0	1	0	
	1	0	0	1	0	0	0	1	1	0	0	
	1	0	0	1	0	0	0	1	1	1	0	

	1	0	0	1	0	0	1	0	0	0	0	
	1	0	0	1	0	0	1	0	0	1	0	
	1	0	0	1	0	0	1	0	1	0	0	
	1	0	0	1	0	0	1	0	1	1	0	
	1	0	0	1	0	0	1	1	0	0	0	
	1	0	0	1	0	0	1	1	0	1	0	
	1	0	0	1	0	0	1	1	1	0	0	
	1	0	0	1	0	0	1	1	1	1	0	
	1	0	0	1	0	1	0	0	0	0	0	
	1	0	0	1	0	1	0	0	0	1	0	
	1	0	0	1	0	1	0	0	1	0	0	
	1	0	0	1	0	1	0	0	1	1	0	
	1	0	0	1	0	1	0	1	0	0	0	
	1	0	0	1	0	1	0	1	0	1	0	
	1	0	0	1	0	1	0	1	1	0	0	
	1	0	0	1	0	1	0	1	1	1	0	
	1	0	0	1	0	1	1	0	0	0	0	
	1	0	0	1	0	1	1	0	0	1	0	
	1	0	0	1	0	1	1	0	1	0	0	
	1	0	0	1	0	1	1	0	1	1	0	
	1	0	0	1	0	1	1	1	0	0	0	
	1	0	0	1	0	1	1	1	0	1	0	
	1	0	0	1	0	1	1	1	1	0	0	
	1	0	0	1	0	1	1	1	1	1	0	
	1	0	0	1	1	0	0	0	0	0	0	
	1	0	0	1	1	0	0	0	0	1	0	
	1	0	0	1	1	0	0	0	1	0	0	
	1	0	0	1	1	0	0	0	1	1	0	
	1	0	0	1	1	0	0	1	0	0	0	
	1	0	0	1	1	0	0	1	0	1	0	
	1	0	0	1	1	0	0	1	1	0	0	
	1	0	0	1	1	0	0	1	1	1	0	
58	1	0	0	1	1	0	1	0	0	0	0	CREB or MITF is required for the transcriptional activation of Bcl-2 gene.<br>Phosphorylation of BAD (a Bcl-2 antagonist) by Akt, ERK, or RSK is needed for the full activation of Bcl-2 protein.<br>Beta-catenin and GSK3-beta upregulates Bcl-2 expression level.<br>Bcl-2 activity is suppressed if any of its negative regulators is activated.<br>Hence, the activation condition for Bcl-2_M is 'AND(OR(MITFprotein_M, CREB_M), OR(Akt_M, ERK_M, RSK_M, b-catenin_M, GSK3b_M), NOT(OR(p53_M, JNK_M, p38_M)))'.
	1	0	0	1	1	0	1	0	0	1	0	
	1	0	0	1	1	0	1	0	1	0	0	
	1	0	0	1	1	0	1	0	1	1	0	
	1	0	0	1	1	0	1	1	0	0	0	
	1	0	0	1	1	0	1	1	0	1	0	
	1	0	0	1	1	0	1	1	1	0	0	
	1	0	0	1	1	0	1	1	1	1	0	
	1	0	0	1	1	1	0	0	0	0	0	
	1	0	0	1	1	1	0	0	0	1	0	
	1	0	0	1	1	1	0	0	1	0	0	
	1	0	0	1	1	1	0	0	1	1	0	
	1	0	0	1	1	1	0	1	0	0	0	
	1	0	0	1	1	1	0	1	0	1	0	
	1	0	0	1	1	1	0	1	1	0	0	
	1	0	0	1	1	1	0	1	1	1	0	
	1	0	0	1	1	1	1	0	0	0	0	
	1	0	0	1	1	1	1	0	0	1	0	
	1	0	0	1	1	1	1	0	1	0	0	
	1	0	0	1	1	1	1	0	1	1	0	
	1	0	0	1	1	1	1	1	0	0	0	
	1	0	0	1	1	1	1	1	0	1	0	
	1	0	0	1	1	1	1	1	1	0	0	
	1	0	0	1	1	1	1	1	1	1	0	
	1	0	1	0	0	0	0	0	0	0	0	
	1	0	1	0	0	0	0	0	0	1	0	
	1	0	1	0	0	0	0	0	1	0	0	
	1	0	1	0	0	0	0	0	1	1	0	
	1	0	1	0	0	0	0	1	0	0	0	
	1	0	1	0	0	0	0	1	0	1	0	
	1	0	1	0	0	0	0	1	1	0	0	
	1	0	1	0	0	0	0	1	1	1	0	
	1	0	1	0	0	0	1	0	0	0	0	

	1	0	1	0	0	0	1	0	0	1	0
	1	0	1	0	0	0	1	0	1	0	0
	1	0	1	0	0	0	1	0	1	1	0
	1	0	1	0	0	0	1	1	0	0	0
	1	0	1	0	0	0	1	1	0	1	0
	1	0	1	0	0	0	1	1	1	0	0
	1	0	1	0	0	0	1	1	1	1	0
	1	0	1	0	0	1	0	0	0	0	0
	1	0	1	0	0	1	0	0	0	1	0
	1	0	1	0	0	1	0	0	1	0	0
	1	0	1	0	0	1	0	0	1	1	0
	1	0	1	0	0	1	0	1	0	0	0
	1	0	1	0	0	1	0	1	0	1	0
	1	0	1	0	0	1	0	1	1	0	0
	1	0	1	0	0	1	0	1	1	1	0
	1	0	1	0	0	1	1	0	0	0	0
	1	0	1	0	0	1	1	0	0	1	0
	1	0	1	0	0	1	1	0	1	0	0
	1	0	1	0	0	1	1	0	1	1	0
	1	0	1	0	0	1	1	1	0	0	0
	1	0	1	0	0	1	1	1	0	1	0
	1	0	1	0	0	1	1	1	1	0	0
	1	0	1	0	0	1	1	1	1	1	0
	1	0	1	0	1	0	0	0	0	0	0
	1	0	1	0	1	0	0	0	0	1	0
	1	0	1	0	1	0	0	0	1	0	0
	1	0	1	0	1	0	0	0	1	1	0
	1	0	1	0	1	0	0	1	0	0	0
	1	0	1	0	1	0	0	1	0	1	0
	1	0	1	0	1	0	0	1	1	0	0
	1	0	1	0	1	0	0	1	1	1	0
	1	0	1	0	1	0	1	0	0	0	0
58	1	0	1	0	1	0	1	0	0	1	0
	1	0	1	0	1	0	1	0	1	0	0
	1	0	1	0	1	0	1	0	1	1	0
	1	0	1	0	1	0	1	1	0	0	0
	1	0	1	0	1	0	1	1	0	1	0
	1	0	1	0	1	0	1	1	1	0	0
	1	0	1	0	1	0	1	1	1	1	0
	1	0	1	0	1	1	0	0	0	0	0
	1	0	1	0	1	1	0	0	0	1	0
	1	0	1	0	1	1	0	0	1	0	0
	1	0	1	0	1	1	0	0	1	1	0
	1	0	1	0	1	1	0	1	0	0	0
	1	0	1	0	1	1	0	1	0	1	0
	1	0	1	0	1	1	0	1	1	0	0
	1	0	1	0	1	1	0	1	1	1	0
	1	0	1	0	1	1	1	0	0	0	0
	1	0	1	0	1	1	1	0	0	1	0
	1	0	1	0	1	1	1	0	1	0	0
	1	0	1	0	1	1	1	0	1	1	0
	1	0	1	0	1	1	1	1	0	0	0
	1	0	1	0	1	1	1	1	0	1	0
	1	0	1	0	1	1	1	1	1	0	0
	1	0	1	0	1	1	1	1	1	1	0
	1	0	1	1	0	0	0	0	0	0	0
	1	0	1	1	0	0	0	0	0	1	0
	1	0	1	1	0	0	0	0	1	0	0
	1	0	1	1	0	0	0	0	1	1	0
	1	0	1	1	0	0	0	1	0	0	0
	1	0	1	1	0	0	0	1	0	1	0
	1	0	1	1	0	0	0	1	1	0	0
	1	0	1	1	0	0	0	1	1	1	0
	1	0	1	1	0	0	1	0	0	0	0
	1	0	1	1	0	0	1	0	0	1	0

CREB or MITF is required for the transcriptional activation of Bcl-2 gene.
Phosphorylation of BAD (a Bcl-2 antagonist) by Akt, ERK, or RSK is needed for the full activation of Bcl-2 protein.
Beta-catenin and GSK3-beta upregulates Bcl-2 expression level.
Bcl-2 activity is suppressed if any of its negative regulators is activated.
Hence, the activation condition for Bcl-2_M is 'AND(OR(MITFprotein_M, CREB_M), OR(Akt_M, ERK_M, RSK_M, b-catenin_M, GSK3b_M), NOT(OR(p53_M, JNK_M, p38_M)))'.

	1	0	1	1	0	0	1	0	1	0	0
	1	0	1	1	0	0	1	0	1	1	0
	1	0	1	1	0	0	1	1	0	0	0
	1	0	1	1	0	0	1	1	0	1	0
	1	0	1	1	0	0	1	1	1	0	0
	1	0	1	1	0	0	1	1	1	1	0
	1	0	1	1	0	1	0	0	0	0	0
	1	0	1	1	0	1	0	0	0	1	0
	1	0	1	1	0	1	0	0	1	0	0
	1	0	1	1	0	1	0	0	1	1	0
	1	0	1	1	0	1	0	1	0	0	0
	1	0	1	1	0	1	0	1	0	1	0
	1	0	1	1	0	1	0	1	1	0	0
	1	0	1	1	0	1	0	1	1	1	0
	1	0	1	1	0	1	1	0	0	0	0
	1	0	1	1	0	1	1	0	0	1	0
	1	0	1	1	0	1	1	0	1	0	0
	1	0	1	1	0	1	1	0	1	1	0
	1	0	1	1	0	1	1	1	0	0	0
	1	0	1	1	0	1	1	1	0	1	0
	1	0	1	1	0	1	1	1	1	0	0
	1	0	1	1	0	1	1	1	1	1	0
	1	0	1	1	1	0	0	0	0	0	0
	1	0	1	1	1	0	0	0	0	1	0
	1	0	1	1	1	0	0	0	1	0	0
	1	0	1	1	1	0	0	0	1	1	0
	1	0	1	1	1	0	0	1	0	0	0
	1	0	1	1	1	0	0	1	0	1	0
	1	0	1	1	1	0	0	1	1	0	0
	1	0	1	1	1	0	0	1	1	1	0
	1	0	1	1	1	0	1	0	0	0	0
	1	0	1	1	1	0	1	0	0	1	0
58	1	0	1	1	1	0	1	0	1	0	0
	1	0	1	1	1	0	1	0	1	1	0
	1	0	1	1	1	0	1	1	0	0	0
	1	0	1	1	1	0	1	1	0	1	0
	1	0	1	1	1	0	1	1	1	0	0
	1	0	1	1	1	0	1	1	1	1	0
	1	0	1	1	1	1	0	0	0	0	0
	1	0	1	1	1	1	0	0	0	1	0
	1	0	1	1	1	1	0	0	1	0	0
	1	0	1	1	1	1	0	0	1	1	0
	1	0	1	1	1	1	0	1	0	0	0
	1	0	1	1	1	1	0	1	0	1	0
	1	0	1	1	1	1	0	1	1	0	0
	1	0	1	1	1	1	0	1	1	1	0
	1	0	1	1	1	1	1	0	0	0	0
	1	0	1	1	1	1	1	0	0	1	0
	1	0	1	1	1	1	1	0	1	0	0
	1	0	1	1	1	1	1	0	1	1	0
	1	0	1	1	1	1	1	1	0	0	0
	1	0	1	1	1	1	1	1	0	1	0
	1	0	1	1	1	1	1	1	1	0	0
	1	0	1	1	1	1	1	1	1	1	0
	1	1	0	0	0	0	0	0	0	0	0
	1	1	0	0	0	0	0	0	0	1	0
	1	1	0	0	0	0	0	0	1	0	0
	1	1	0	0	0	0	0	0	1	1	0
	1	1	0	0	0	0	0	1	0	0	0
	1	1	0	0	0	0	0	1	0	1	0
	1	1	0	0	0	0	0	1	1	0	0
	1	1	0	0	0	0	0	1	1	1	0
	1	1	0	0	0	0	1	0	0	0	0
	1	1	0	0	0	0	1	0	0	1	0
	1	1	0	0	0	0	1	0	1	0	0

CREB or MITF is required for the transcriptional activation of Bcl-2 gene.
Phosphorylation of BAD (a Bcl-2 antagonist) by Akt, ERK, or RSK is needed for the full activation of Bcl-2 protein.
Beta-catenin and GSK3-beta upregulates Bcl-2 expression level.
Bcl-2 activity is suppressed if any of its negative regulators is activated.
Hence, the activation condition for Bcl-2_M is 'AND(OR(MITFprotein_M, CREB_M), OR(Akt_M, ERK_M, RSK_M, b-catenin_M, GSK3b_M), NOT(OR(p53_M, JNK_M, p38_M)))'.

	1	1	0	0	0	0	1	0	1	1	0	
	1	1	0	0	0	0	1	1	0	0	0	
	1	1	0	0	0	0	1	1	0	1	0	
	1	1	0	0	0	0	1	1	1	0	0	
	1	1	0	0	0	0	1	1	1	1	0	
	1	1	0	0	0	1	0	0	0	0	0	
	1	1	0	0	0	1	0	0	0	1	0	
	1	1	0	0	0	1	0	0	1	0	0	
	1	1	0	0	0	1	0	0	1	1	0	
	1	1	0	0	0	1	0	1	0	0	0	
	1	1	0	0	0	1	0	1	0	1	0	
	1	1	0	0	0	1	0	1	1	0	0	
	1	1	0	0	0	1	0	1	1	1	0	
	1	1	0	0	0	1	1	0	0	0	0	
	1	1	0	0	0	1	1	0	0	1	0	
	1	1	0	0	0	1	1	0	1	0	0	
	1	1	0	0	0	1	1	0	1	1	0	
	1	1	0	0	0	1	1	1	0	0	0	
	1	1	0	0	0	1	1	1	0	1	0	
	1	1	0	0	0	1	1	1	1	0	0	
	1	1	0	0	0	1	1	1	1	1	0	
	1	1	0	0	1	0	0	0	0	0	0	
	1	1	0	0	1	0	0	0	0	1	0	
	1	1	0	0	1	0	0	0	1	0	0	
	1	1	0	0	1	0	0	0	1	1	0	
	1	1	0	0	1	0	0	1	0	0	0	
	1	1	0	0	1	0	0	1	0	1	0	
	1	1	0	0	1	0	0	1	1	0	0	
	1	1	0	0	1	0	0	1	1	1	0	
	1	1	0	0	1	0	1	0	0	0	0	
	1	1	0	0	1	0	1	0	0	1	0	
	1	1	0	0	1	0	1	0	1	0	0	
58	1	1	0	0	1	0	1	0	1	1	0	CREB or MITF is required for the transcriptional activation of Bcl-2 gene.<br>Phosphorylation of BAD (a Bcl-2 antagonist) by Akt, ERK, or RSK is needed for the full activation of Bcl-2 protein.<br>Beta-catenin and GSK3-beta upregulates Bcl-2 expression level.<br>Bcl-2 activity is suppressed if any of its negative regulators is activated.<br>Hence, the activation condition for Bcl-2_M is 'AND(OR(MITFprotein_M, CREB_M), OR(Akt_M, ERK_M, RSK_M, b-catenin_M, GSK3b_M), NOT(OR(p53_M, JNK_M, p38_M)))'.
	1	1	0	0	1	0	1	1	0	0	0	
	1	1	0	0	1	0	1	1	0	1	0	
	1	1	0	0	1	0	1	1	1	0	0	
	1	1	0	0	1	0	1	1	1	1	0	
	1	1	0	0	1	1	0	0	0	0	0	
	1	1	0	0	1	1	0	0	0	1	0	
	1	1	0	0	1	1	0	0	1	0	0	
	1	1	0	0	1	1	0	0	1	1	0	
	1	1	0	0	1	1	0	1	0	0	0	
	1	1	0	0	1	1	0	1	0	1	0	
	1	1	0	0	1	1	0	1	1	0	0	
	1	1	0	0	1	1	0	1	1	1	0	
	1	1	0	0	1	1	1	0	0	0	0	
	1	1	0	0	1	1	1	0	0	1	0	
	1	1	0	0	1	1	1	0	1	0	0	
	1	1	0	0	1	1	1	0	1	1	0	
	1	1	0	0	1	1	1	1	0	0	0	
	1	1	0	0	1	1	1	1	0	1	0	
	1	1	0	0	1	1	1	1	1	0	0	
	1	1	0	0	1	1	1	1	1	1	0	
	1	1	0	1	0	0	0	0	0	0	0	
	1	1	0	1	0	0	0	0	0	1	0	
	1	1	0	1	0	0	0	0	1	0	0	
	1	1	0	1	0	0	0	0	1	1	0	
	1	1	0	1	0	0	0	1	0	0	0	
	1	1	0	1	0	0	0	1	0	1	0	
	1	1	0	1	0	0	0	1	1	0	0	
	1	1	0	1	0	0	0	1	1	1	0	
	1	1	0	1	0	0	1	0	0	0	0	
	1	1	0	1	0	0	1	0	0	1	0	
	1	1	0	1	0	0	1	0	1	0	0	
	1	1	0	1	0	0	1	0	1	1	0	

	1	1	0	1	0	0	1	1	0	0	0	
	1	1	0	1	0	0	1	1	0	1	0	
	1	1	0	1	0	0	1	1	1	0	0	
	1	1	0	1	0	0	1	1	1	1	0	
	1	1	0	1	0	1	0	0	0	0	0	
	1	1	0	1	0	1	0	0	0	1	0	
	1	1	0	1	0	1	0	0	1	0	0	
	1	1	0	1	0	1	0	0	1	1	0	
	1	1	0	1	0	1	0	1	0	0	0	
	1	1	0	1	0	1	0	1	0	1	0	
	1	1	0	1	0	1	0	1	1	0	0	
	1	1	0	1	0	1	0	1	1	1	0	
	1	1	0	1	0	1	1	0	0	0	0	
	1	1	0	1	0	1	1	0	0	1	0	
	1	1	0	1	0	1	1	0	1	0	0	
	1	1	0	1	0	1	1	0	1	1	0	
	1	1	0	1	0	1	1	1	0	0	0	
	1	1	0	1	0	1	1	1	0	1	0	
	1	1	0	1	0	1	1	1	1	0	0	
	1	1	0	1	0	1	1	1	1	1	0	
	1	1	0	1	1	0	0	0	0	0	0	
	1	1	0	1	1	0	0	0	0	1	0	
	1	1	0	1	1	0	0	0	1	0	0	
	1	1	0	1	1	0	0	0	1	1	0	
	1	1	0	1	1	0	0	1	0	0	0	
	1	1	0	1	1	0	0	1	0	1	0	
	1	1	0	1	1	0	0	1	1	0	0	
	1	1	0	1	1	0	0	1	1	1	0	
	1	1	0	1	1	0	1	0	0	0	0	
	1	1	0	1	1	0	1	0	0	1	0	
	1	1	0	1	1	0	1	0	1	0	0	
	1	1	0	1	1	0	1	0	1	1	0	
58	1	1	0	1	1	0	1	1	0	0	0	CREB or MITF is required for the transcriptional activation of Bcl-2 gene.<br>Phosphorylation of BAD (a Bcl-2 antagonist) by Akt, ERK, or RSK is needed for the full activation of Bcl-2 protein.<br>Beta-catenin and GSK3-beta upregulates Bcl-2 expression level.<br>Bcl-2 activity is suppressed if any of its negative regulators is activated.<br>Hence, the activation condition for Bcl-2_M is 'AND(OR(MITFprotein_M, CREB_M), OR(Akt_M, ERK_M, RSK_M, b-catenin_M, GSK3b_M), NOT(OR(p53_M, JNK_M, p38_M)))'.
	1	1	0	1	1	0	1	1	0	1	0	
	1	1	0	1	1	0	1	1	1	0	0	
	1	1	0	1	1	0	1	1	1	1	0	
	1	1	0	1	1	1	0	0	0	0	0	
	1	1	0	1	1	1	0	0	0	1	0	
	1	1	0	1	1	1	0	0	1	0	0	
	1	1	0	1	1	1	0	0	1	1	0	
	1	1	0	1	1	1	0	1	0	0	0	
	1	1	0	1	1	1	0	1	0	1	0	
	1	1	0	1	1	1	0	1	1	0	0	
	1	1	0	1	1	1	0	1	1	1	0	
	1	1	0	1	1	1	1	0	0	0	0	
	1	1	0	1	1	1	1	0	0	1	0	
	1	1	0	1	1	1	1	0	1	0	0	
	1	1	0	1	1	1	1	0	1	1	0	
	1	1	0	1	1	1	1	1	0	0	0	
	1	1	0	1	1	1	1	1	0	1	0	
	1	1	0	1	1	1	1	1	1	0	0	
	1	1	0	1	1	1	1	1	1	1	0	
	1	1	1	0	0	0	0	0	0	0	0	
	1	1	1	0	0	0	0	0	0	1	0	
	1	1	1	0	0	0	0	0	1	0	0	
	1	1	1	0	0	0	0	0	1	1	0	
	1	1	1	0	0	0	0	1	0	0	0	
	1	1	1	0	0	0	0	1	0	1	0	
	1	1	1	0	0	0	0	1	1	0	0	
	1	1	1	0	0	0	0	1	1	1	0	
	1	1	1	0	0	0	1	0	0	0	0	
	1	1	1	0	0	0	1	0	0	1	0	
	1	1	1	0	0	0	1	0	1	0	0	
	1	1	1	0	0	0	1	0	1	1	0	
	1	1	1	0	0	0	1	1	0	0	0	

	1	1	1	0	0	0	1	1	0	1	0	
	1	1	1	0	0	0	1	1	1	0	0	
	1	1	1	0	0	0	1	1	1	1	0	
	1	1	1	0	0	1	0	0	0	0	0	
	1	1	1	0	0	1	0	0	0	1	0	
	1	1	1	0	0	1	0	0	1	0	0	
	1	1	1	0	0	1	0	0	1	1	0	
	1	1	1	0	0	1	0	1	0	0	0	
	1	1	1	0	0	1	0	1	0	1	0	
	1	1	1	0	0	1	0	1	1	0	0	
	1	1	1	0	0	1	0	1	1	1	0	
	1	1	1	0	0	1	1	0	0	0	0	
	1	1	1	0	0	1	1	0	0	1	0	
	1	1	1	0	0	1	1	0	1	0	0	
	1	1	1	0	0	1	1	0	1	1	0	
	1	1	1	0	0	1	1	1	0	0	0	
	1	1	1	0	0	1	1	1	0	1	0	
	1	1	1	0	0	1	1	1	1	0	0	
	1	1	1	0	0	1	1	1	1	1	0	
	1	1	1	0	1	0	0	0	0	0	0	
	1	1	1	0	1	0	0	0	0	1	0	
	1	1	1	0	1	0	0	0	1	0	0	
	1	1	1	0	1	0	0	0	1	1	0	
	1	1	1	0	1	0	0	1	0	0	0	
	1	1	1	0	1	0	0	1	0	1	0	
	1	1	1	0	1	0	0	1	1	0	0	
	1	1	1	0	1	0	0	1	1	1	0	
	1	1	1	0	1	0	1	0	0	0	0	
	1	1	1	0	1	0	1	0	0	1	0	
	1	1	1	0	1	0	1	0	1	0	0	
	1	1	1	0	1	0	1	0	1	1	0	
	1	1	1	0	1	0	1	1	0	0	0	
58	1	1	1	0	1	0	1	1	0	1	0	CREB or MITF is required for the transcriptional activation of Bcl-2 gene.<br>Phosphorylation of BAD (a Bcl-2 antagonist) by Akt, ERK, or RSK is needed for the full activation of Bcl-2 protein.<br>Beta-catenin and GSK3-beta upregulates Bcl-2 expression level.<br>Bcl-2 activity is suppressed if any of its negative regulators is activated.<br>Hence, the activation condition for Bcl-2_M is 'AND(OR(MITFprotein_M, CREB_M), OR(Akt_M, ERK_M, RSK_M, b-catenin_M, GSK3b_M), NOT(OR(p53_M, JNK_M, p38_M)))'.
	1	1	1	0	1	0	1	1	1	0	0	
	1	1	1	0	1	0	1	1	1	1	0	
	1	1	1	0	1	1	0	0	0	0	0	
	1	1	1	0	1	1	0	0	0	1	0	
	1	1	1	0	1	1	0	0	1	0	0	
	1	1	1	0	1	1	0	0	1	1	0	
	1	1	1	0	1	1	0	1	0	0	0	
	1	1	1	0	1	1	0	1	0	1	0	
	1	1	1	0	1	1	0	1	1	0	0	
	1	1	1	0	1	1	0	1	1	1	0	
	1	1	1	0	1	1	1	0	0	0	0	
	1	1	1	0	1	1	1	0	0	1	0	
	1	1	1	0	1	1	1	0	1	0	0	
	1	1	1	0	1	1	1	0	1	1	0	
	1	1	1	0	1	1	1	1	0	0	0	
	1	1	1	0	1	1	1	1	0	1	0	
	1	1	1	0	1	1	1	1	1	0	0	
	1	1	1	0	1	1	1	1	1	1	0	
	1	1	1	1	0	0	0	0	0	0	0	
	1	1	1	1	0	0	0	0	0	1	0	
	1	1	1	1	0	0	0	0	1	0	0	
	1	1	1	1	0	0	0	0	1	1	0	
	1	1	1	1	0	0	0	1	0	0	0	
	1	1	1	1	0	0	0	1	0	1	0	
	1	1	1	1	0	0	0	1	1	0	0	
	1	1	1	1	0	0	0	1	1	1	0	
	1	1	1	1	0	0	1	0	0	0	0	
	1	1	1	1	0	0	1	0	0	1	0	
	1	1	1	1	0	0	1	0	1	0	0	
	1	1	1	1	0	0	1	0	1	1	0	
	1	1	1	1	0	0	1	1	0	0	0	
	1	1	1	1	0	0	1	1	0	1	0	

58

1	1	1	1	0	0	1	1	1	0	0
1	1	1	1	0	0	1	1	1	1	0
1	1	1	1	0	1	0	0	0	0	0
1	1	1	1	0	1	0	0	0	1	0
1	1	1	1	0	1	0	0	1	0	0
1	1	1	1	0	1	0	0	1	1	0
1	1	1	1	0	1	0	1	0	0	0
1	1	1	1	0	1	0	1	0	1	0
1	1	1	1	0	1	0	1	1	0	0
1	1	1	1	0	1	0	1	1	1	0
1	1	1	1	0	1	1	0	0	0	0
1	1	1	1	0	1	1	0	0	1	0
1	1	1	1	0	1	1	0	1	0	0
1	1	1	1	0	1	1	0	1	1	0
1	1	1	1	0	1	1	1	0	0	0
1	1	1	1	0	1	1	1	0	1	0
1	1	1	1	0	1	1	1	1	0	0
1	1	1	1	0	1	1	1	1	1	0
1	1	1	1	1	0	0	0	0	0	0
1	1	1	1	1	0	0	0	0	1	0
1	1	1	1	1	0	0	0	1	0	0
1	1	1	1	1	0	0	0	1	1	0
1	1	1	1	1	0	0	1	0	0	0
1	1	1	1	1	0	0	1	0	1	0
1	1	1	1	1	0	0	1	1	0	0
1	1	1	1	1	0	0	1	1	1	0
1	1	1	1	1	0	1	0	0	0	0
1	1	1	1	1	0	1	0	0	1	0
1	1	1	1	1	0	1	0	1	0	0
1	1	1	1	1	0	1	0	1	1	0
1	1	1	1	1	0	1	1	0	0	0
1	1	1	1	1	0	1	1	0	1	0
1	1	1	1	1	0	1	1	1	0	0
1	1	1	1	1	0	1	1	1	1	0
1	1	1	1	1	1	0	0	0	0	0
1	1	1	1	1	1	0	0	0	1	0
1	1	1	1	1	1	0	0	1	0	0
1	1	1	1	1	1	0	0	1	1	0
1	1	1	1	1	1	0	1	0	0	0
1	1	1	1	1	1	0	1	0	1	0
1	1	1	1	1	1	0	1	1	0	0
1	1	1	1	1	1	0	1	1	1	0
1	1	1	1	1	1	1	0	0	0	0
1	1	1	1	1	1	1	0	0	1	0
1	1	1	1	1	1	1	0	1	0	0
1	1	1	1	1	1	1	0	1	1	0
1	1	1	1	1	1	1	1	0	0	0
1	1	1	1	1	1	1	1	0	1	0
1	1	1	1	1	1	1	1	1	0	0
1	1	1	1	1	1	1	1	1	1	0

CREB or MITF is required for the transcriptional activation of Bcl-2 gene.
Phosphorylation of BAD (a Bcl-2 antagonist) by Akt, ERK, or RSK is needed for the full activation of Bcl-2 protein.
Beta-catenin and GSK3-beta upregulates Bcl-2 expression level.
Bcl-2 activity is suppressed if any of its negative regulators is activated.
Hence, the activation condition for Bcl-2_M is 'AND(OR(MITFprotein_M, CREB_M), OR(Akt_M, ERK_M, RSK_M, b-catenin_M, GSK3b_M), NOT(OR(p53_M, JNK_M, p38_M)))'.

59

B-catenin_M	CREB_M	MITFmRNA_M
0	0	0
0	1	0
1	0	0
1	1	1

Binding of beta-catenin and CREB to the MITF gene promoter region stimulates MITF transcription, hence forming an AND relation.

60	MITFmRNA_M, ERK_M → MITFprotein_M<br>0 0 → 0<br>0 1 → 0<br>1 0 → 0<br>1 1 → 1	The phosphorylation of MITF at serine 73 by ERK results in upregulation of the MITF melanogenic function.
61	MITFprotein_M → Melanin<br>0 → 0<br>1 → 1	MITF promotes melanin synthesis by upregulating expression of melanogenic enzymes.

Blue and red boxes denote positive and negative regulators, respectively.

Supplementary Table S3. ***In silico*** **node control anaysis for the identification of appropriate strategies to reduce UVB-induced skin pigmentation.**

Perturbed node	Δ Melanin (%)		Δ Bcl-2_K (%)		Δ Bcl-2_M (%)	
	Perturbation		Perturbation		Perturbation	
	(-)	(+)	(-)	(+)	(-)	(+)
Akt_K	24.84	-57.02	-29.80	45.62	14.22	-70.77
a-MSH_K	37.27	-1.14	2.46	-0.75	17.23	-41.21
ASK1_K	-13.95	31.73	42.15	-100.00	-17.88	-11.96
b-catenin_K	0.00	-4.34	0.00	0.46	0.00	-5.86
COX-2_K	9.82	-27.99	-0.44	0.50	18.40	-44.35
EGFR_K	13.49	-57.03	-31.75	45.62	3.44	-70.77
ERK_K	-6.00	29.44	-2.67	-56.84	-6.40	-7.69
ET-1_K	-60.72	371.98	2.45	36.92	-71.30	178.15
GSK3b_K	-27.99	9.82	0.50	-0.44	-44.35	18.40
IL-1_K	-78.43	316.83	8.13	26.75	-71.61	151.76
JNK_K	0.00	0.00	0.00	-72.95	0.00	0.00
MDM2_K	10.24	-14.47	-4.44	11.13	5.82	-29.37
MEK_K	-6.00	30.85	-2.67	-46.15	-6.40	-5.97
MKK4_K	0.00	0.00	0.00	-72.95	0.00	0.00
MKK6_K	-13.95	31.73	28.74	-100.00	-17.88	-11.96
NFAT_K	0.00	-4.34	0.00	0.46	0.00	-5.86
p38_K	-13.95	30.43	28.74	-100.00	-17.88	-12.77
p53_K	-14.49	62.81	11.12	-70.93	-29.38	-3.30
PDK1_K	24.94	-57.01	-29.78	45.62	14.24	-70.76
PGE2_K	9.82	0.81	-0.44	-0.40	18.40	-40.30
PI3K_K	24.93	-57.03	-29.76	45.62	14.34	-70.78
PTEN_K	-10.43	24.93	13.28	-29.76	-11.58	14.34
RAF_K	-6.00	30.85	-2.67	-46.15	-6.40	-5.97
RAS_K	-6.00	-1.00	-2.67	-23.78	-6.40	-15.48
RSK_K	0.00	0.00	-5.35	6.17	0.00	0.00
SCF_K	-36.46	34.92	1.31	0.08	-34.08	29.66
SG_K	-6.00	-0.68	-2.67	-23.78	-6.40	-15.30
AC_M	37.35	1.19	2.45	-0.31	17.25	-40.40
Akt_M	-100.00	157.75	7.78	9.77	-100.00	189.53
ASK1_M	77.58	-100.00	-1.04	7.78	120.00	-100.00
b-catenin_M	-100.00	81.02	7.78	-2.07	-8.15	1.82
cAMP_M	51.07	-100.00	3.99	7.78	28.74	-100.00
c-kit_M	-36.46	34.92	1.31	0.08	-34.08	29.66
CREB_M	-100.00	6.97	7.78	0.18	-86.62	1.09
EP4_M	9.82	1.19	-0.44	-0.31	18.40	-40.40
ERK_M	-100.00	118.89	7.78	-3.56	-58.53	0.54

ETR_M	-60.72	372.69	2.45	37.08	-71.30	178.48
GSK3b_M	81.02	-100.00	-2.07	7.78	1.82	-13.75
JNK_M	0.00	0.00	0.00	0.00	0.00	-100.00
MC1R_M	37.35	0.81	2.45	-0.40	17.25	-40.30
MDM2_M	0.00	0.00	0.00	0.00	-22.55	15.48
MEK_M	-100.00	9.19	7.78	0.52	-58.53	5.21
MITFmRNA_M	-100.00	95.71	7.78	-1.02	-23.75	1.58
MITFprotein_M	-100.00	402.49	7.78	45.62	-23.75	-100.00
MKK4_M	0.00	0.00	0.00	0.00	0.00	-100.00
MKK6_M	77.62	-100.00	-1.10	7.78	91.15	-100.00
MSK_M	-10.72	6.97	0.23	0.18	-7.26	1.09
p38_M	77.70	-100.00	-1.29	7.78	91.35	-100.00
p53_M	0.00	0.00	0.00	0.00	15.48	-100.00
PDE_M	-35.54	51.07	2.45	3.99	-27.01	28.74
PDK1_M	-100.00	157.75	7.78	9.77	-100.00	189.53
PI3K_M	-100.00	157.19	7.78	9.84	-100.00	189.53
PKA_M	-42.82	51.38	2.83	4.00	-34.06	31.92
PKC_M	-60.73	372.69	2.45	37.08	-71.30	178.48
RAF_M	-100.00	9.11	7.78	0.54	-58.53	5.30
RAS_M	-52.83	372.69	1.77	37.08	-49.26	178.48
RSK_M	0.00	0.00	0.00	0.00	-15.61	17.09
SG_M	-36.46	372.69	1.31	37.08	-34.08	178.48

Node control analysis for the identification of appropriate strategies to reduce UVB-induced skin pigmentation. Each internal regulatory node was pinned to either ‘0’ or ‘1’ before Boolean model simulations were performed under UVB stimulation. The perturbation effect of the node control of each internal regulatory node was measured as described in the Materials and Methods. '-' and '+' denote inhibition and constitutive activation, respectively.

In the model**,** activation of MITFprotein_M requires activation of both ERK_M and MITFmRNA_M, and activation of MITFmRNA_M requires activation of both CREB_M and b-catenin_M. Therefore, in the model simulation, inhibition of either ERK_M or MITFmRNA_M results in inactivation of MITFprotein_M, which leads to 100% Melanin reduction. Similarly, inhibition of either CREB_M or b-catenin_M results in inactivation of MITFmRNA_M, which causes inactivation of MITFprotein_M and consequently 100% Melanin reduction. In conclusion, activation of Melanin node requires activation of ERK_M, CREB_M, beta-catenin_M, MITFmRNA_M, and MITFprotein_M nodes, and therefore inhibition of any of these nodes results in 100% Melanin reduction in the model simulation.

Supplementary Table S4. *In silico* analysis of changes in UVB-induced melanin synthesis with respect to the inhibition of beta-catenin, Ras, PKA, or a combination of Ras and PKA.

Node #1 perturbation	Node #2 perturbation	Δ Melanin (%)
beta-catenin inhibition	-	-100.00
Ras inhibition	-	-52.83
PKA inhibition	-	-42.82
Ras inhibition	PKA inhibition	-24.05

Inhibition of beta-catenin, Ras, or PKA can reduce the activity of node 'melanin' in response to UVB irradiation. Among these three intervention strategies, the most effective strategy was beta-catenin inhibition. The simultaneous inhibition of Ras and PKA was less effective in suppressing the melanin synthesis compared to the individual inhibition of any of these nodes. See Fig. S4 for the biochemical validation of the simulation results.

III. Supplementary References

1 Peus, D. *et al.* H2O2 is an important mediator of UVB-induced EGF-receptor phosphorylation in cultured keratinocytes. *J Invest Dermatol* **110**, 966-971, (1998).

2 Hsieh, C. C. & Papaconstantinou, J. Thioredoxin-ASK1 complex levels regulate ROS-mediated p38 MAPK pathway activity in livers of aged and long-lived Snell dwarf mice. *Faseb J* **20**, 259-268, (2006).

3 Van Laethem, A. *et al.* Apoptosis signal regulating kinase-1 connects reactive oxygen species to p38 MAPK-induced mitochondrial apoptosis in UVB-irradiated human keratinocytes. *Free radical biology & medicine* **41**, 1361-1371, (2006).

4 Kupper, T. S., Chua, A. O., Flood, P., McGuire, J. & Gubler, U. Interleukin 1 gene expression in cultured human keratinocytes is augmented by ultraviolet irradiation. *The Journal of clinical investigation* **80**, 430-436, (1987).

5 Lee, S. R. *et al.* Reversible inactivation of the tumor suppressor PTEN by H2O2. *The Journal of biological chemistry* **277**, 20336-20342, (2002).

6 Ming, M. *et al.* UVB-induced ERK/AKT-dependent PTEN suppression promotes survival of epidermal keratinocytes. *Oncogene* **29**, 492-502, (2010).

7 Lemmon, M. A. & Schlessinger, J. Cell signaling by receptor tyrosine kinases. *Cell* **141**, 1117-1134, (2010).

8 Kim, A. H., Khursigara, G., Sun, X., Franke, T. F. & Chao, M. V. Akt phosphorylates and negatively regulates apoptosis signal-regulating kinase 1. *Mol Cell Biol* **21**, 893-901, (2001).

9 Hu, Y. & Bowtell, D. D. Sos1 rapidly associates with Grb2 and is hypophosphorylated when complexed with the EGF receptor after EGF stimulation. *Oncogene* **12**, 1865-1872 (1996).

10 Rao, G. N. Hydrogen peroxide induces complex formation of SHC-Grb2-SOS with receptor tyrosine kinase and activates Ras and extracellular signal-regulated protein kinases group of

mitogen-activated protein kinases. *Oncogene* **13**, 713-719 (1996).

11 Sebastian, S. *et al.* The complexity of targeting EGFR signalling in cancer: from expression to turnover. *Biochimica et biophysica acta* **1766**, 120-139, (2006).

12 Cherniack, A. D., Klarlund, J. K., Conway, B. R. & Czech, M. P. Disassembly of Son-of-sevenless proteins from Grb2 during p21ras desensitization by insulin. *The Journal of biological chemistry* **270**, 1485-1488 (1995).

13 Scheid, M. P. & Woodgett, J. R. Unravelling the activation mechanisms of protein kinase B/Akt. *Febs Lett* **546**, 108-112 (2003).

14 Toker, A. & Newton, A. C. Cellular signaling: pivoting around PDK-1. *Cell* **103**, 185-188 (2000).

15 Song, M. S., Salmena, L. & Pandolfi, P. P. The functions and regulation of the PTEN tumour suppressor. *Nature reviews. Molecular cell biology* **13**, 283-296, (2012).

16 Matsukawa, J., Matsuzawa, A., Takeda, K. & Ichijo, H. The ASK1-MAP kinase cascades in mammalian stress response. *Journal of biochemistry* **136**, 261-265, (2004).

17 Park, H. S. *et al.* Akt (protein kinase B) negatively regulates SEK1 by means of protein phosphorylation. *The Journal of biological chemistry* **277**, 2573-2578, (2002).

18 Chardin, P. *et al.* Human Sos1: a guanine nucleotide exchange factor for Ras that binds to GRB2. *Science* **260**, 1338-1343 (1993).

19 Wang, X., McCullough, K. D., Franke, T. F. & Holbrook, N. J. Epidermal growth factor receptor-dependent Akt activation by oxidative stress enhances cell survival. *The Journal of biological chemistry* **275**, 14624-14631 (2000).

20 Derijard, B. *et al.* Independent human MAP-kinase signal transduction pathways defined by MEK and MKK isoforms. *Science* **267**, 682-685 (1995).

21 Zimmermann, S. & Moelling, K. Phosphorylation and regulation of Raf by Akt (protein kinase B). *Science* **286**, 1741-1744 (1999).

22 Jelinek, T., Dent, P., Sturgill, T. W. & Weber, M. J. Ras-induced activation of Raf-1 is dependent on tyrosine phosphorylation. *Mol Cell Biol* **16**, 1027-1034 (1996).

23 Cross, D. A., Alessi, D. R., Cohen, P., Andjelkovich, M. & Hemmings, B. A. Inhibition of glycogen synthase kinase-3 by insulin mediated by protein kinase B. *Nature* **378**, 785-789, (1995).

24 Hart, M. J., de los Santos, R., Albert, I. N., Rubinfeld, B. & Polakis, P. Downregulation of beta-catenin by human Axin and its association with the APC tumor suppressor, beta-catenin and GSK3 beta. *Current biology : CB* **8**, 573-581 (1998).

25 Howe, L. R. *et al.* Activation of the MAP kinase pathway by the protein kinase raf. *Cell* **71**, 335-342 (1992).

26 Kyriakis, J. M. *et al.* Raf-1 activates MAP kinase-kinase. *Nature* **358**, 417-421, (1992).

27 Alessi, D. R. *et al.* Identification of the sites in MAP kinase kinase-1 phosphorylated by p74raf-1. *Embo J* **13**, 1610-1619 (1994).

28 Mayo, L. D. & Donner, D. B. A phosphatidylinositol 3-kinase/Akt pathway promotes translocation of Mdm2 from the cytoplasm to the nucleus. *Proc Natl Acad Sci U S A* **98**, 11598-11603, (2001).

29 Beals, C. R., Sheridan, C. M., Turck, C. W., Gardner, P. & Crabtree, G. R. Nuclear export of NF-ATc enhanced by glycogen synthase kinase-3. *Science* **275**, 1930-1934 (1997).

30 Neal, J. W. & Clipstone, N. A. Glycogen synthase kinase-3 inhibits the DNA binding activity of NFATc. *The Journal of biological chemistry* **276**, 3666-3673, (2001).

31 Araki, Y. *et al.* Regulation of cyclooxygenase-2 expression by the Wnt and ras pathways. *Cancer research* **63**, 728-734 (2003).

32 Tang, Q., Gonzales, M., Inoue, H. & Bowden, G. T. Roles of Akt and glycogen synthase kinase 3beta in the ultraviolet B induction of cyclooxygenase-2 transcription in human keratinocytes. *Cancer research* **61**, 4329-4332 (2001).

33 Flockhart, R. J., Diffey, B. L., Farr, P. M., Lloyd, J. & Reynolds, N. J. NFAT regulates induction of COX-2 and apoptosis of keratinocytes in response to ultraviolet radiation exposure. *Faseb J* **22**, 4218-4227, (2008).

34 Chen, W., Tang, Q., Gonzales, M. S. & Bowden, G. T. Role of p38 MAP kinases and ERK in mediating ultraviolet-B induced cyclooxygenase-2 gene expression in human keratinocytes.

Oncogene **20**, 3921-3926, (2001).

35 Robinson, M. J. & Cobb, M. H. Mitogen-activated protein kinase pathways. *Current opinion in cell biology* **9**, 180-186 (1997).

36 Schlessinger, J. Cell signaling by receptor tyrosine kinases. *Cell* **103**, 211-225 (2000).

37 Zhang, H., Shi, X., Hampong, M., Blanis, L. & Pelech, S. Stress-induced inhibition of ERK1 and ERK2 by direct interaction with p38 MAP kinase. *The Journal of biological chemistry* **276**, 6905-6908, (2001).

38 Li, S. P., Junttila, M. R., Han, J., Kahari, V. M. & Westermarck, J. p38 Mitogen-activated protein kinase pathway suppresses cell survival by inducing dephosphorylation of mitogen-activated protein/extracellular signal-regulated kinase kinase1,2. *Cancer research* **63**, 3473-3477 (2003).

39 Melnikova, V. O., Santamaria, A. B., Bolshakov, S. V. & Ananthaswamy, H. N. Mutant p53 is constitutively phosphorylated at Serine 15 in UV-induced mouse skin tumors: involvement of ERK1/2 MAP kinase. *Oncogene* **22**, 5958-5966, (2003).

40 Honda, R., Tanaka, H. & Yasuda, H. Oncoprotein MDM2 is a ubiquitin ligase E3 for tumor suppressor p53. *Febs Lett* **420**, 25-27 (1997).

41 Bulavin, D. V. *et al.* Phosphorylation of human p53 by p38 kinase coordinates N-terminal phosphorylation and apoptosis in response to UV radiation. *Embo J* **18**, 6845-6854, (1999).

42 Dalby, K. N., Morrice, N., Caudwell, F. B., Avruch, J. & Cohen, P. Identification of regulatory phosphorylation sites in mitogen-activated protein kinase (MAPK)-activated protein kinase-1a/p90rsk that are inducible by MAPK. *The Journal of biological chemistry* **273**, 1496-1505 (1998).

43 She, Q. B., Ma, W. Y., Zhong, S. & Dong, Z. Activation of JNK1, RSK2, and MSK1 is involved in serine 112 phosphorylation of Bad by ultraviolet B radiation. *The Journal of biological chemistry* **277**, 24039-24048, (2002).

44 Datta, S. R. *et al.* Akt phosphorylation of BAD couples survival signals to the cell-intrinsic death machinery. *Cell* **91**, 231-241 (1997).

45 Pugazhenthi, S. *et al.* Akt/protein kinase B up-regulates Bcl-2 expression through cAMP-response element-binding protein. *The Journal of biological chemistry* **275**, 10761-10766 (2000).

46 Breitschopf, K., Haendeler, J., Malchow, P., Zeiher, A. M. & Dimmeler, S. Posttranslational modification of Bcl-2 facilitates its proteasome-dependent degradation: molecular characterization of the involved signaling pathway. *Mol Cell Biol* **20**, 1886-1896 (2000).

47 Yamamoto, K., Ichijo, H. & Korsmeyer, S. J. BCL-2 is phosphorylated and inactivated by an ASK1/Jun N-terminal protein kinase pathway normally activated at G(2)/M. *Mol Cell Biol* **19**, 8469-8478 (1999).

48 Sunayama, J., Tsuruta, F., Masuyama, N. & Gotoh, Y. JNK antagonizes Akt-mediated survival (2005).

49 Li, Q., Dashwood, W. M., Zhong, X., Nakagama, H. & Dashwood, R. H. Bcl-2 overexpression in PhIP-induced colon tumors: cloning of the rat Bcl-2 promoter and characterization of a pathway involving beta-catenin, c-Myc and E2F1. *Oncogene* **26**, 6194-6202, (2007).

50 Dickey, A. *et al.* GSK-3beta inhibition promotes cell death, apoptosis, and in vivo tumor growth delay in neuroblastoma Neuro-2A cell line. *Journal of neuro-oncology* **104**, 145-153, (2011).

51 Yun, S. I., Yoon, H. Y. & Chung, Y. S. Glycogen synthase kinase-3beta regulates etoposide-induced apoptosis via Bcl-2 mediated caspase-3 activation in C3H10T1/2 cells. *Apoptosis : an international journal on programmed cell death* **14**, 771-777, (2009).

52 De Chiara, G. *et al.* Bcl-2 Phosphorylation by p38 MAPK: identification of target sites and biologic consequences. *The Journal of biological chemistry* **281**, 21353-21361, (2006).

53 Miyashita, T., Harigai, M., Hanada, M. & Reed, J. C. Identification of a p53-dependent negative response element in the bcl-2 gene. *Cancer research* **54**, 3131-3135 (1994).

54 Haldar, S., Negrini, M., Monne, M., Sabbioni, S. & Croce, C. M. Down-regulation of bcl-2 by p53 in breast cancer cells. *Cancer research* **54**, 2095-2097 (1994).

55 Miyashita, T. *et al.* Tumor suppressor p53 is a regulator of bcl-2 and bax gene expression in vitro and in vivo. *Oncogene* **9**, 1799-1805 (1994).

56 Ballif, B. A. & Blenis, J. Molecular mechanisms mediating mammalian mitogen-activated protein kinase (MAPK) kinase (MEK)-MAPK cell survival signals. *Cell growth & differentiation : the molecular biology journal of the American Association for Cancer Research* **12**, 397-408 (2001).

57 Bonni, A. *et al.* Cell survival promoted by the Ras-MAPK signaling pathway by transcription-dependent and -independent mechanisms. *Science* **286**, 1358-1362 (1999).

58 Imokawa, G., Yada, Y. & Miyagishi, M. Endothelins secreted from human keratinocytes are intrinsic mitogens for human melanocytes. *The Journal of biological chemistry* **267**, 24675-24680 (1992).

59 Murase, D. *et al.* The essential role of p53 in hyperpigmentation of the skin via regulation of paracrine melanogenic cytokine receptor signaling. *The Journal of biological chemistry* **284**, 4343-4353, (2009).

60 Hyter, S. *et al.* Endothelin-1 is a transcriptional target of p53 in epidermal keratinocytes and regulates ultraviolet-induced melanocyte homeostasis. *Pigment cell & melanoma research* **26**, 247-258, (2013).

61 Hachiya, A. *et al.* Biphasic expression of two paracrine melanogenic cytokines, stem cell factor and endothelin-1, in ultraviolet B-induced human melanogenesis. *The American journal of pathology* **165**, 2099-2109, (2004).

62 Buckman, S. Y. *et al.* COX-2 expression is induced by UVB exposure in human skin: implications for the development of skin cancer. *Carcinogenesis* **19**, 723-729 (1998).

63 Grewe, M. *et al.* Analysis of the mechanism of ultraviolet (UV) B radiation-induced prostaglandin E2 synthesis by human epidermoid carcinoma cells. *J Invest Dermatol* **101**, 528-531 (1993).

64 Tada, A., Pereira, E., Beitner-Johnson, D., Kavanagh, R. & Abdel-Malek, Z. A. Mitogen- and ultraviolet-B-induced signaling pathways in normal human melanocytes. *J Invest Dermatol* **118**, 316-322, (2002).

65 Hachiya, A., Kobayashi, A., Ohuchi, A., Takema, Y. & Imokawa, G. The paracrine role of stem cell factor/c-kit signaling in the activation of human melanocytes in ultraviolet-B-induced

pigmentation. *J Invest Dermatol* **116**, 578-586, (2001).

66 Thommes, K., Lennartsson, J., Carlberg, M. & Ronnstrand, L. Identification of Tyr-703 and Tyr-936 as the primary association sites for Grb2 and Grb7 in the c-Kit/stem cell factor receptor. *Biochem J* **341 (Pt 1)**, 211-216 (1999).

67 D'Orazio, J. A. *et al.* Topical drug rescue strategy and skin protection based on the role of Mc1r in UV-induced tanning. *Nature* **443**, 340-344, (2006).

68 Kabashima, K. *et al.* Prostaglandin E2 is required for ultraviolet B-induced skin inflammation via EP2 and EP4 receptors. *Laboratory investigation; a journal of technical methods and pathology* **87**, 49-55, (2007).

69 Lennartsson, J. *et al.* Phosphorylation of Shc by Src family kinases is necessary for stem cell factor receptor/c-kit mediated activation of the Ras/MAP kinase pathway and c-fos induction. *Oncogene* **18**, 5546-5553, (1999).

70 Mountjoy, K. G., Robbins, L. S., Mortrud, M. T. & Cone, R. D. The cloning of a family of genes that encode the melanocortin receptors. *Science* **257**, 1248-1251 (1992).

71 Price, E. R. *et al.* alpha-Melanocyte-stimulating hormone signaling regulates expression of microphthalmia, a gene deficient in Waardenburg syndrome. *The Journal of biological chemistry* **273**, 33042-33047 (1998).

72 Walser, R. *et al.* PKCbeta phosphorylates PI3Kgamma to activate it and release it from GPCR control. *PLoS biology* **11**, e1001587, (2013).

73 Rodriguez-Viciana, P. *et al.* Phosphatidylinositol-3-OH kinase as a direct target of Ras. *Nature* **370**, 527-532, (1994).

74 Khaled, M. *et al.* Glycogen synthase kinase 3beta is activated by cAMP and plays an active role in the regulation of melanogenesis. *The Journal of biological chemistry* **277**, 33690-33697, (2002).

75 Busca, R. *et al.* Ras mediates the cAMP-dependent activation of extracellular signal-regulated kinases (ERKs) in melanocytes. *Embo J* **19**, 2900-2910, (2000).

76 Garcia-Borron, J. C., Sanchez-Laorden, B. L. & Jimenez-Cervantes, C. Melanocortin-1 receptor

structure and functional regulation. *Pigment cell research / sponsored by the European Society for Pigment Cell Research and the International Pigment Cell Society* **18**, 393-410, (2005).

77 Conti, M. & Beavo, J. Biochemistry and physiology of cyclic nucleotide phosphodiesterases: essential components in cyclic nucleotide signaling. *Annual review of biochemistry* **76**, 481-511, (2007).

78 Omori, K. & Kotera, J. Overview of PDEs and their regulation. *Circulation research* **100**, 309-327, (2007).

79 Kolch, W. *et al.* Protein kinase C alpha activates RAF-1 by direct phosphorylation. *Nature* **364**, 249-252, (1993).

80 Sette, C. & Conti, M. Phosphorylation and activation of a cAMP-specific phosphodiesterase by the cAMP-dependent protein kinase. Involvement of serine 54 in the enzyme activation. *The Journal of biological chemistry* **271**, 16526-16534 (1996).

81 MacKenzie, S. J. *et al.* Long PDE4 cAMP specific phosphodiesterases are activated by protein kinase A-mediated phosphorylation of a single serine residue in Upstream Conserved Region 1 (UCR1). *British journal of pharmacology* **136**, 421-433, (2002).

82 McCoy, C. E., Campbell, D. G., Deak, M., Bloomberg, G. B. & Arthur, J. S. MSK1 activity is controlled by multiple phosphorylation sites. *Biochem J* **387**, 507-517, (2005).

83 Giuliano, S. *et al.* Microphthalmia-associated transcription factor controls the DNA damage response and a lineage-specific senescence program in melanomas. *Cancer research* **70**, 3813-3822, (2010).

84 Du, K. & Montminy, M. CREB is a regulatory target for the protein kinase Akt/PKB. *The Journal of biological chemistry* **273**, 32377-32379 (1998).

85 Pugazhenthi, S. *et al.* Insulin-like growth factor I-mediated activation of the transcription factor cAMP response element-binding protein in PC12 cells. Involvement of p38 mitogen-activated protein kinase-mediated pathway. *The Journal of biological chemistry* **274**, 2829-2837 (1999).

86 McGill, G. G. *et al.* Bcl2 regulation by the melanocyte master regulator Mitf modulates lineage

survival and melanoma cell viability. *Cell* **109**, 707-718 (2002).

87 Schepsky, A. *et al.* The microphthalmia-associated transcription factor Mitf interacts with beta-catenin to determine target gene expression. *Mol Cell Biol* **26**, 8914-8927, (2006).

88 Mizutani, Y., Hayashi, N., Kawashima, M. & Imokawa, G. A single UVB exposure increases the expression of functional KIT in human melanocytes by up-regulating MITF expression through the phosphorylation of p38/CREB. *Archives of dermatological research* **302**, 283-294, (2010).

89 Hemesath, T. J., Price, E. R., Takemoto, C., Badalian, T. & Fisher, D. E. MAP kinase links the transcription factor Microphthalmia to c-Kit signalling in melanocytes. *Nature* **391**, 298-301, (1998).

90 Price, E. R. *et al.* Lineage-specific signaling in melanocytes. C-kit stimulation recruits p300/CBP to microphthalmia. *The Journal of biological chemistry* **273**, 17983-17986 (1998).

91 Sato, S. *et al.* CBP/p300 as a co-factor for the Microphthalmia transcription factor. *Oncogene* **14**, 3083-3092, (1997).

92 Lin, J. Y. & Fisher, D. E. Melanocyte biology and skin pigmentation. *Nature* **445**, 843-850, (2007).
